# Joint effects of pregnancy, sociocultural, and environmental factors on early life gut microbiome structure and diversity

**DOI:** 10.1038/srep31775

**Published:** 2016-08-25

**Authors:** Albert M. Levin, Alexandra R. Sitarik, Suzanne L. Havstad, Kei E. Fujimura, Ganesa Wegienka, Andrea E. Cassidy-Bushrow, Haejin Kim, Edward M. Zoratti, Nicholas W. Lukacs, Homer A. Boushey, Dennis R. Ownby, Susan V. Lynch, Christine C. Johnson

**Affiliations:** 1 Department of Public Health Sciences, Henry Ford Health System, Detroit, MI, 48202, USA; 2 Center for Bioinformatics, Henry Ford Health System, Detroit, MI, 48202, USA; 3 Division of Gastroenterology, Department of Medicine, University of California, San Francisco, CA, 94143, USA; 4 Division of Allergy and Clinical Immunology, Department of Medicine, Henry Ford Health System, Detroit, MI, 48202, USA; 5 Department of Pathology, University of Michigan, Ann Arbor, MI, 48109, USA; 6 Division of Pulmonary and Critical Care Medicine, Department of Medicine, University of California, San Francisco, CA, 94143, USA; 7 Division of Allergy and Immunology, Medical College of Georgia at Augusta University, Augusta, GA, 30912, USA.

## Abstract

The joint impact of pregnancy, environmental, and sociocultural exposures on early life gut microbiome is not yet well-characterized, especially in racially and socioeconomically diverse populations. Gut microbiota of 298 children from a Detroit-based birth cohort were profiled using 16S rRNA sequencing: 130 neonates (median age = 1.2 months) and 168 infants (median age = 6.6 months). Multiple factors were associated with neonatal gut microbiome composition in both single- and multi-factor models, with independent contributions of maternal race-ethnicity, breastfeeding, mode of delivery, marital status, exposure to environmental tobacco smoke, and indoor pets. These findings were consistent in the infants, and networks demonstrating the shared impact of factors on gut microbial composition also showed notable topological similarity between neonates and infants. Further, latent groups defined by these factors explained additional variation, highlighting the importance of combinatorial effects. Our findings also have implications for studies investigating the impact of the early life gut microbiota on disease.

The human gut microbiome, the mixed-species community of microbes that reside in the gastrointestinal tract, plays a critical role in physiological and immunological maturation and homeostasis^1^. Perturbations to gut bacterial community composition in neonatancy have been associated with a variety of pediatric disorders^1^,^2^,^3^,^4^,^5^,^6^, underscoring the relationship between early life gut microbiota development and childhood health status. Many environmental and social risk factors associated with disease or health may also influence early life gut microbial development. However, only a few studies have examined relationships between early life exposures and the gut microbiota^7^,^8^,^9^. While these studies may have examined multiple pregnancy, sociocultural, and environmental factors, none have taken a multi-factor approach to studying their joint effects on gut microbiota composition, and none have been conducted in a racially and socioeconomically diverse birth cohort.

To date, the most comprehensive study associating pregnancy and environmental exposures the with early life gut microbiome was conducted by Bäckhed *et al*.^7^, where longitudinal samples from 98 Swedish mother-infant dyads were examined. This study and others have confirmed that early feeding patterns have a dramatic effect on the infant’s developing gut microbial community composition^10^,^11^, as have delivery mode and antibiotic exposure^12^,^13^,^14^. If the influence of these and potentially other early life exposures on disease risk is thought to be partially explained by their impact on gut microbiome development over the first year of life, a broader understanding of such factors and their influence on bacterial community composition is critical.

In the current study, we used 16S rRNA sequencing to profile the bacterial gut microbiota present in stool specimens gathered during the first year of life in a racially and socioeconomically diverse population-based birth cohort from the metropolitan Detroit area. Associations between a broad survey of pre- and post-natal environmental and sociocultural factors and early life gut microbiome composition were evaluated both individually and in multivariable models to identify factors that influence this critical period of microbial assemblage. Networks of associated factors were constructed to visualize the relationships between these factors based on shared compositional impact. We also explored how groupings of mothers based on these factors may identify possible combined effects that further explain the early life gut microbiota composition of their offspring.

## Results

### Early life gut microbiome structure in the Microbes, Asthma, Allergy, and Pets (MAAP) study

**Figure 1:**
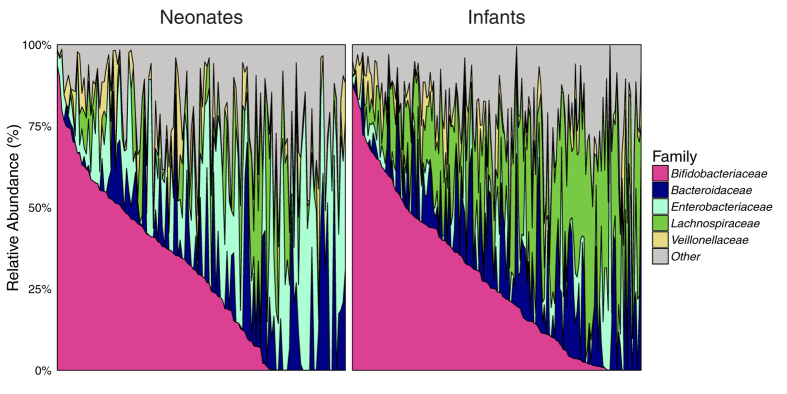
Gut bacterial composition among neonates (N = 130) and infants (N = 168). Relative abundances are displayed at the family level for the five most abundant families, with each vertical spike representing a stacked bar plot for each individual child. The “neonates” are those subjects with specimen collection targeted for the 1-month study visit (actual age range: 0.5–4.6 months, median: 1.2 months), while the “infants” had specimens collected at the study visit targeted for 6-months (age range: 5.6–10.6 months, median: 6.6 months). For each group, subjects are displayed in decreasing order of relative abundance of *Bifidobacteriaceae*.

The MAAP study is derived from the Wayne County Health Environment Asthma and Allergy Longitudinal Study (WHEALS) birth cohort according to study inclusion and exclusion criteria (Supplementary Fig. S1). Of the 298 independent MAAP study subjects with stool specimens available for analysis following sequence quality control filtering, 130 were collected at the study visit targeted for 1-month (actual visit date in months: min = 0.5; 25^th^ percentile = 0.9; median = 1.2; 75^th^ percentile = 1.5; max = 4.6), collectively referred to as “neonates”, and 168 were collected at the study visit targeted for 6-months (actual visit date in months: min = 5.6; 25^th^ percentile = 6.1; median = 6.6; 75^th^ percentile = 7.4; max = 10.6), collectively referred to as “infants”. As expected from previous studies^15^, the gut microbiota exhibited substantial age-related taxonomic variation. In neonatal specimens, bacterial communities were typically dominated by *Bifidobacteriaceae* or *Enterobacteriaceae* taxa (Fig. 1). In comparison, infants were characteristically dominated either by *Bifidobacteriaceae* or *Lachnospiraceae* (Fig. 1), the latter representing a common dominant family in adult gut microbiomes^16^.

**Figure 2:**
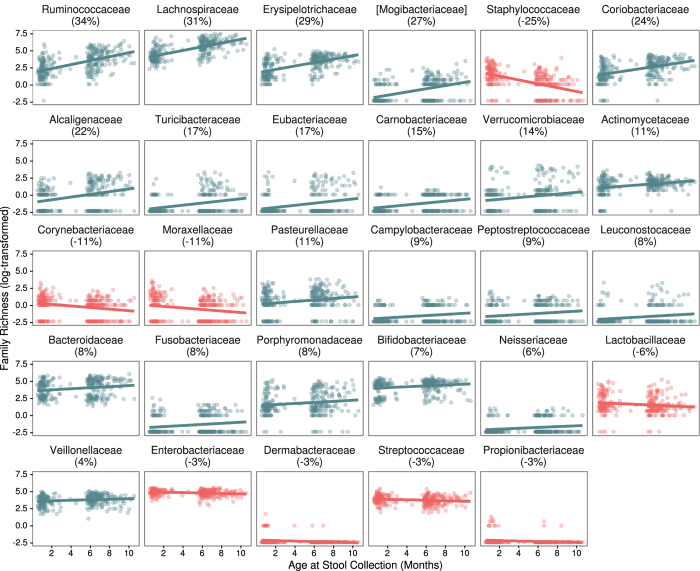
Within-family richness by age at specimen collection. All families with a significant trend (FDR adjusted p-value < 0.05) of richness (the number of unique taxa present for a particular family) with age at stool specimen collection across all 298 subjects are displayed. Families are ordered (left to right and then down) by absolute effect size, shown in parentheses under each family name. Effect sizes can be interpreted as the percent change in the number of unique taxa present in each family for a 1-month increase in age at stool collection. The color indicates direction of association (blue = increasing richness with increasing age and red = decreasing richness with increasing age).

In addition, 21 specific families exhibited taxonomic expansion (i.e. increased richness) with age (false discovery rate [FDR] adjusted p < 0.05, Fig. 2). These included *Lachnospiraceae, Bifidobacteriaceae, Peptostreptococcaceae*, and *Veillonellaceae*. In contrast, 8 families exhibited reciprocal trends (i.e. decreased richness) with age, which included *Enterobacteriaceae, Staphylococcaceae*, and *Streptococcaceae*. These results are consistent with data indicating that the pioneering species that initially colonize the nascent gastrointestinal tract are facultative anaerobes, which are replaced by strict anaerobic taxa as the microbial burden increases, oxygen availability becomes limiting^17^, and the consortium shifts to fermentative metabolism^15^.

### Single-factor associations with early life gut microbiota composition

**Figure 3:**
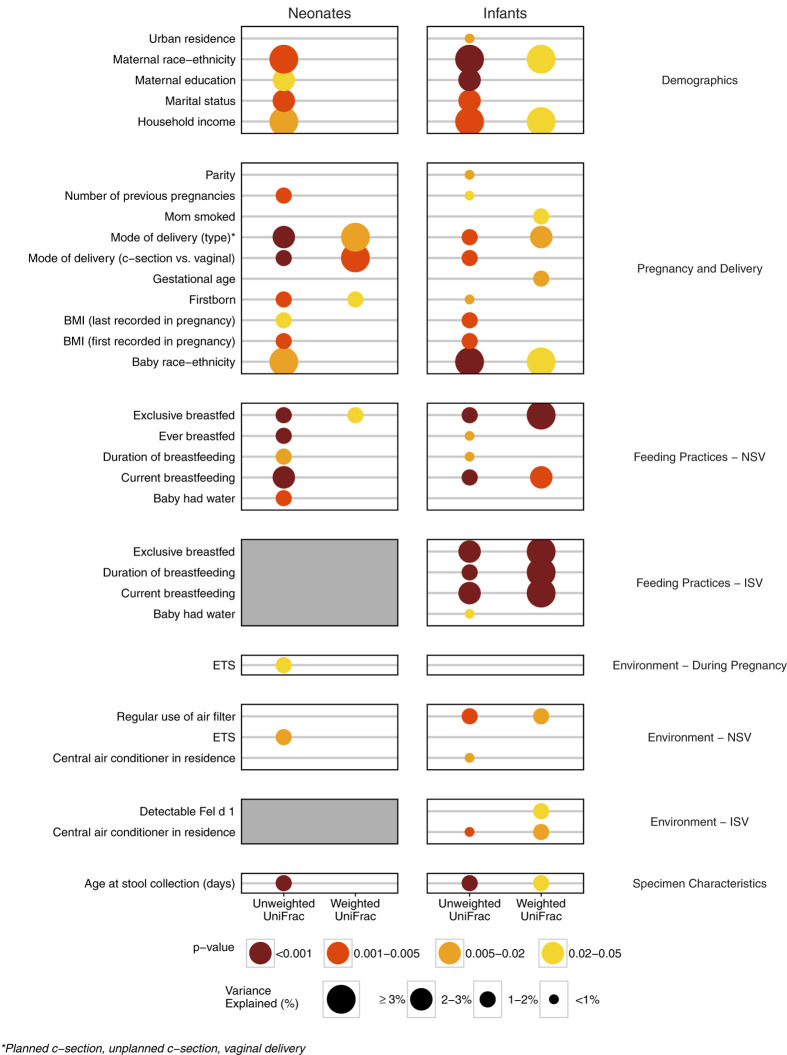
Single-factor gut microbiome compositional analyses for both neonates and infants. Single-factor PERMANOVA composition models were constructed independently for neonates and infants (and by unweighted and weighted UniFrac distance metrics). Only those factors that were significantly associated with composition (p-value < 0.05) in at least one of the four models are displayed. Abbreviations: BMI, body mass index; ETS, environmental tobacco smoke; NSV, neonatal study visit at 1-month of age; ISV, infant study visit at 6-months of age.

Permutational multivariate analysis of variance (PERMANOVA)^18^ was used to test for compositional differences by early life factors, using both unweighted and weighted UniFrac distance metrics. Among the factors tested, 19 of 49 (39%) were significantly associated with gut microbiome composition in the neonates, while 28 of 72 (39%) were significant in the infants (Fig. 3; Supplementary Table S3); 17 of the 19 factors (90%) associated with neonatal gut bacterial communities were also significant in the infants. For the majority of associated factors, unweighted UniFrac was significant but weighted UniFrac was not, indicating that phylogeny alone was capable of distinguishing composition. However, a select few factors were distinguished by both phylogeny and abundance (i.e. by weighted UniFrac). For both age groups, individual associated factors explained approximately 1–4% of the variation in bacterial microbiome composition. Consistent with the differences in composition between neonates and infants (Fig. 1), the age in days at which the specimen was obtained within each group was significantly associated with composition (unweighted UniFrac p < 0.001 for both neonates and infants).

As anticipated, early life feeding and mode of delivery were associated with gut microbiota composition in both neonates and infants. Besides compositional differences by mode of delivery overall (vaginal vs. C-section), significant differences were observed by type of C-section (planned vs. unplanned) among the infants (weighted UniFrac p = 0.022), indicating that even partial labor may alter microbial composition. Multiple other maternal and pre- and post-natal environmental factors were found to be associated with early life gut microbiota composition. Included among the maternal factors were body mass index (BMI) during pregnancy (unweighted UniFrac in neonates p = 0.004 and in infants p = 0.001) as well as the number of previous pregnancies (unweighted Unifrac in neonates p = 0.004 and in infants p = 0.022). Among the environmental exposures, environmental tobacco smoke (ETS) during pregnancy (unweighted UniFrac p = 0.007) and at the neonatal visit (unweighted UniFrac p = 0.021) were also associated with compositional differences in neonates; community richness and phylogenetic diversity were increased in ETS-exposed relative to unexposed participants (Supplementary Table S4).

Interestingly, maternal reported race-ethnicity was associated with gut bacterial community composition in neonates (unweighted UniFrac p = 0.002) and infants (unweighted UniFrac p < 0.001). African American race-ethnicity was associated with a more rich, even, and phylogenetically diverse gastrointestinal microbiota in both neonates and infants (Supplementary Table S4). In the WHEALS cohort, African American race-ethnicity was positively associated with both urban residence and never married (African American mothers were 47.7% never married and 76.2% from an urban residence, as compared to non-African American mothers who were 13.1% never married and 22.9% from an urban residence, both p < 0.001). Consistently, these two factors were also associated with composition, with similar increases in the alpha diversity measures associated with urban residence and never married (Supplementary Table S4).

### Multi-factor models of neonatal and infant gut microbiome composition

**Figure 4:**
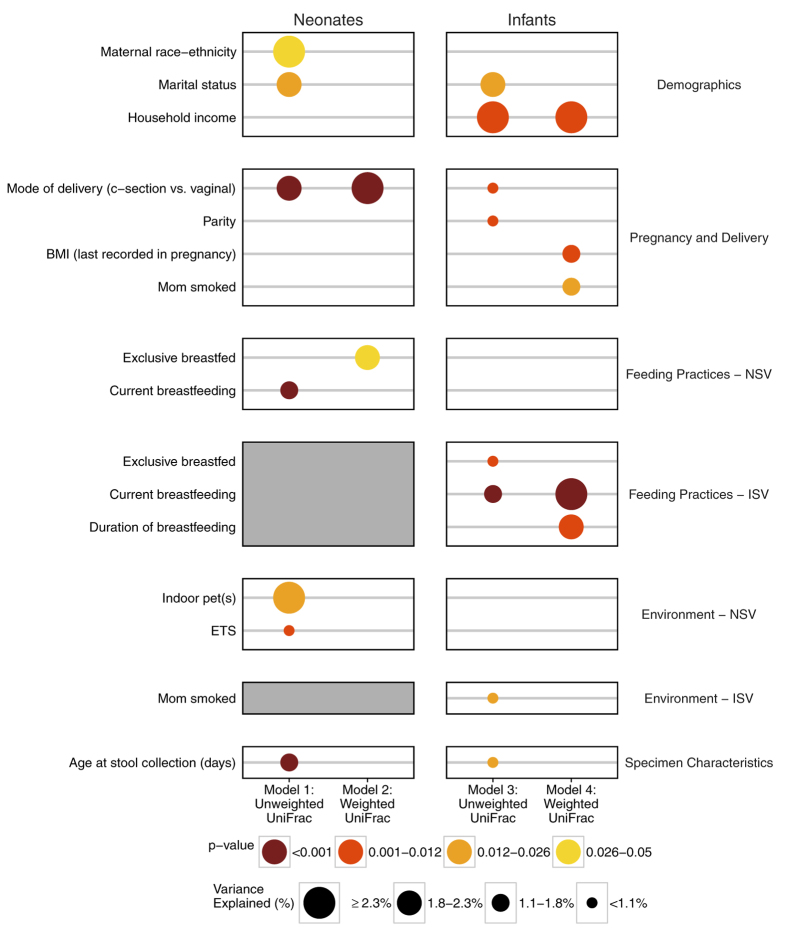
Multi-factor gut microbiome compositional analyses for both neonates and infants. Multi-factor PERMANOVA composition models were constructed independently for neonates and infants (and by unweighted and weighted UniFrac distance metrics) using a backwards variable selection approach. Only factors retained in at least one of the four final multi-factor models are displayed. Abbreviations: BMI, body mass index; ETS, environmental tobacco smoke; NSV, neonatal study visit at 1-month of age; ISV, infant study visit at 6-months of age.

As many of the single factors may explain an overlapping portion of the early life gut microbiome composition, multi-factor PERMANOVA models of microbiome composition were subsequently constructed using a backward variable selection procedure to assess which factors had significant independent effects. The resulting four models by study visit (neonates and infants) and distance metric (weighted and unweighted UniFrac) are presented in Fig. 4. For the neonates, using an unweighted UniFrac distance-based model, seven factors were retained in the model, which included six factors that were univariately associated with bacterial composition (age at stool collection in days (p < 0.001), current breastfeeding at the neonatal visit (p < 0.001), mode of delivery (p < 0.001), ETS exposure at the neonatal visit (p = 0.012), marital status (p = 0.013), and maternal race-ethnicity (p = 0.036)), as well as the presence of an indoor pet(s) (p = 0.022). This model explained 13.9% (adjusted 8.9%) of the variation in the neonatal gut microbiome. The comparable model for the infants similarly retained current breastfeeding (p < 0.001), exclusive breastfeeding (p = 0.006), parity (p = 0.008), mode of delivery (p = 0.009), household income (p = 0.011), age at stool collection in days (p = 0.013), marital status (p = 0.013), and current maternal smoking (p = 0.022). The complete eight factor model explained 12.1% (adjusted 7.7%) of the gut microbiome composition. Taken together, the multi-factor models reflected the remarkable consistency of the single factor analyses, validating the independent and additive effects of these factors on the developing infant microbiome. Further, they reflect that current report of breastfeeding and exposure to tobacco smoke are more influential than past exposure.

### Taxonomic differences associated with factors retained in the multi-factor models

**Figure 5:**
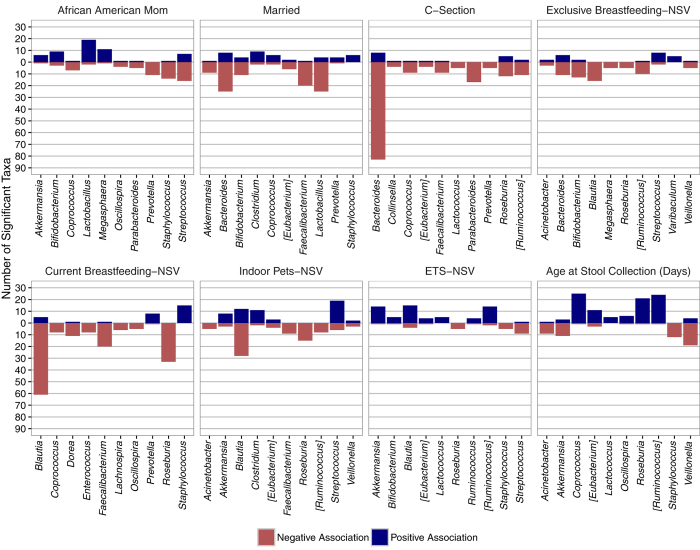
Top genera significantly associated with each factor retained in multi-factor models of neonatal gut microbiome composition. For plotting purposes, “top” genera for each factor were defined using two characteristics: (1) the number of taxa significantly associated with it (to avoid spurious findings) and (2) how “discriminatory” the genera was, defined by consistency in the direction of taxa-specific associations. Each factor displays up to the top ten genera that best discriminated each factor, given the genera had at least 5 significant taxa. Abbreviations: ETS, environmental tobacco smoke; NSV, neonatal study visit at 1-month of age.

**Figure 6:**
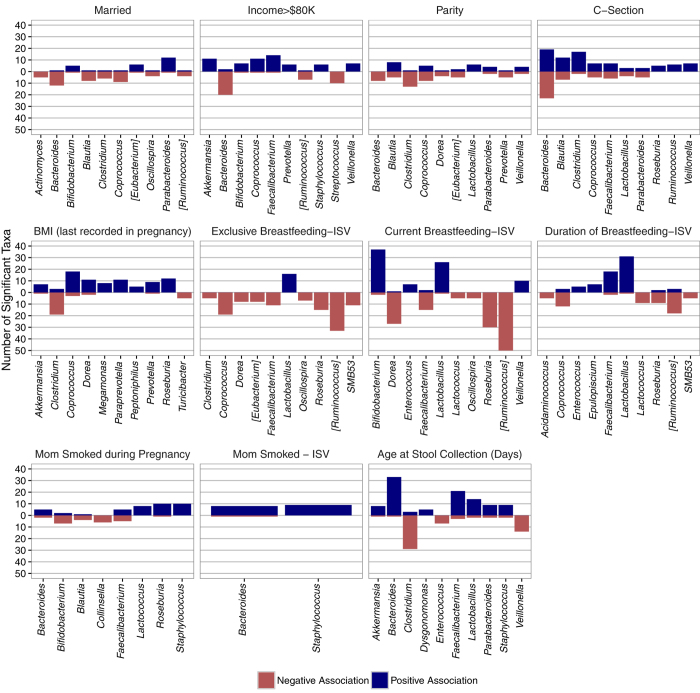
Top genera significantly associated with each factor retained in multi-factor models of infant gut microbiome composition. For plotting purposes, “top” genera for each factor were defined using two characteristics: (1) the number of taxa significantly associated with it (to avoid spurious findings) and (2) how “discriminatory” the genera was, defined by consistency in the direction of taxa-specific associations. Each factor displays up to the top ten genera that best discriminated each factor, given the genera had at least 5 significant taxa. Abbreviations: BMI, body mass index; ISV, infant study visit at 6-months of age.

We next identified how the factors retained in the final multi-factor models associated with differences in taxonomic relative abundances with zero-inflated negative binomial models, adjusting for multiple comparisons using FDR. Discriminant taxa were categorized by genus for both neonates (Fig. 5) and infants (Fig. 6). Among neonates, both current and exclusive breastfeeding were associated with decreased abundance of *Roseburia* taxa; current breastfeeding was associated with an increased abundance of *Staphylococcus* and *Prevotella* taxa, while exclusive breastfeeding was associated with an increased abundance of *Streptococcus* taxa. Collectively, these results are consistent with recent findings from exclusively breastfed 4 month old Swedish infants^7^. Delivery via C-section was primarily characterized by the decreased abundance of specific *Bacteroides, Collinsella*, and *Coprococus* taxa, also consistent with the findings of Bäckhed and colleagues^7^. Compared to all other races, neonates of African American mothers exhibited significantly higher abundances of *Lactobacillus* and *Megasphaera* taxa, while those of married mothers had lower abundances of *Lactobacillus* and *Faecalibacterium* taxa. Neonates currently exposed to ETS had higher abundances of *Ruminococcus* and *Akkermansia* taxa, and those living in a household with an indoor pet(s) were enriched for *Clostridium* taxa and exhibited lower abundances of *Roseburia* taxa.

Among infants (Fig. 6), exclusive breastfeeding was characterized by increased relative abundance of several *Lactobacillus* taxa and decreased abundance of a multitude of taxa from various genera, including *Clostridia, Faecalibacterium* and *Ruminococcus*. Members of these three depleted genera have recently been identified as enriched in older (16–30 month old) Malawian children that (together with 22 other taxa) predict early life chronological age^19^, which is consistent with recent reports indicating that sustained breastfeeding in early life retards the development of an adult-like gut microbiota^7^. A similar trend was observed for current breastfeeding, but the abundance of several *Bifidobacterium* taxa was also increased in addition to *Lactobacillus*. When comparing bacteria impacted by mode of delivery, *Bacteroides* taxa were no longer as clearly enriched in vaginally born children as found with the neonates, which may explain the reduced percentage of variation captured by mode of delivery in these older babies. Infants of married mothers and of high-income households had lower abundances of *Bacteroides* taxa and higher abundances of *Bifidobacterium* taxa. Infants of mothers who smoked either during pregnancy or currently had higher abundances of *Bacteroides* and *Staphylococcus* taxa.

### Networks of factors demonstrating shared impact on gut microbial composition

**Figure 7:**
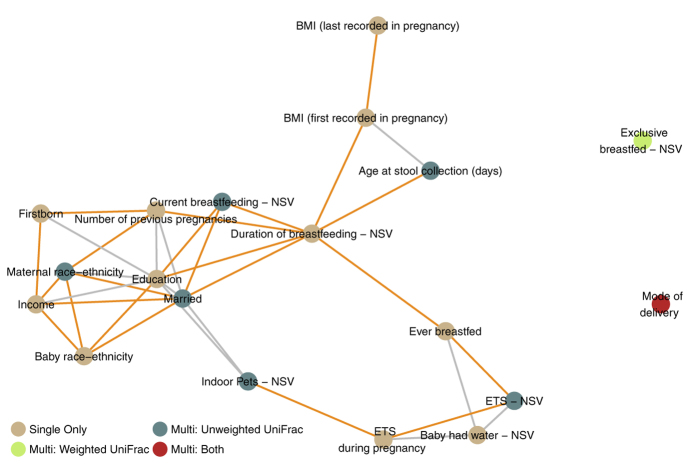
Network of factors demonstrating shared impact on gut microbial composition, among neonates. The network includes factors associated with compositional differences in the neonatal gut microbiome in either single or multi-factor models. Compositional similarity for each pair of factors was defined as the percentage of overlapping significant taxa among all significant taxa between the two factors; two factors were connected in the network if they had at least a 15% overlap in shared associated taxa (≥80^th^ percentile of percentages). In order to disentangle similarity based on compositional impact and similarity due to high correlation, between-factor connections are colored orange if they are significantly correlated (grey otherwise). Abbreviations: BMI, body mass index; ETS, environmental tobacco smoke; NSV, neonatal study visit at 1-month of age.

**Figure 8:**
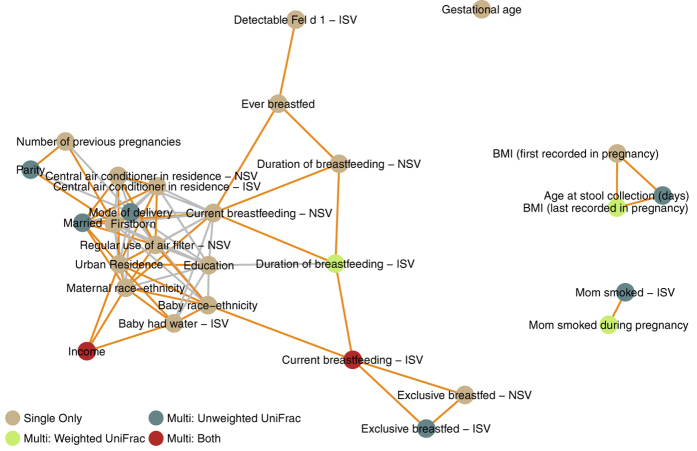
Network of factors demonstrating shared impact on gut microbial composition, among infants. The network includes factors associated with compositional differences in the infant gut microbiome in either single or multi-factor models. Compositional similarity for each pair of factors was defined as the percentage of overlapping significant taxa among all significant taxa between the two factors; two factors were connected in the network if they had at least a 12% overlap in shared associated taxa (≥80^th^ percentile of percentages). In order to disentangle similarity based on compositional impact and similarity due to high correlation, between-factor connections are colored orange if they are significantly correlated (grey otherwise). Abbreviations: BMI, body mass index; NSV, neonatal study visit at 1-month of age; ISV, infant study visit at 6-months of age.

To more concretely interrelate factors with a similar, and statistically significant, impact on bacterial composition, networks of these factors were constructed in neonates (Fig. 7) and infants (Fig. 8), where the distance between pairs of factors was based on the proportion of taxa significantly associated with both (Supplementary Table S5). To help evaluate the extent to which similarity in composition is due to similarity between the factors, pairwise correlations between the factors are also presented in Supplementary Figures S2 and S3 for the neonates and infants, respectively, and significant correlations are highlighted orange between pairs of factors in the corresponding network figures. Among the neonates, maternal race-ethnicity was part of an interconnected module that included socioeconomic (income, marital status, and education), prior pregnancy (firstborn and number of previous pregnancies), and early feeding (current breastfeeding and duration of breastfeeding) factors. ETS factors constituted a separate module, with indoor pet exposure connecting this module with the first. Along with age at stool collection, BMI measures during pregnancy defined a third module, connected with the first via duration of breastfeeding. Both mode of delivery and exclusive breastfeeding were not connected to each other or the broader network, indicating their distinct contributions to bacterial composition in the neonatal gut.

The infant network (Fig. 8) was notably similar in topology to the neonatal network. Maternal race-ethnicity was also part of a highly interconnected module that contained socioeconomic, early feeding, and prior pregnancy factors. This module additionally included housing characteristics (central air conditioner in residence and regular use of air filters). A dissimilarity with the neonatal network was this module’s connection with mode of delivery, indicating a less distinct contribution of mode of delivery in infants compared to neonates. Tobacco smoke exposure and BMI during pregnancy again defined two distinct modules, with BMI during pregnancy again being closely connected to age at stool specimen collection.

In addition to showing the relationships between these factors based on shared compositional impact, these networks also provide insight into the factors selected in the multi-factor models. In general, the modules described were represented in the multi-factor models by one or more of their component factors, the number of which depended upon the complexity of the module. These results also demonstrate that significantly correlated factors may still have distinct effects on composition. For example, among the neonates, maternal race-ethnicity and marital status were significantly correlated with one another, but both were included in the neonatal unweighted UniFrac multi-factor model, indicating significant independent composition contributions.

While the networks demonstrated similarity in topology, differences in the factors selected in the respective multi-factor models were present. In particular, maternal race-ethnicity was part of a module that also contained household income in both neonatal and infant networks; at the same time, maternal race-ethnicity was included in the unweighted UniFrac neonatal model but income was not, while income was included in the unweighted UniFrac infant model but maternal race-ethnicity was not. The consistent grouping of these factors in both early life networks and their mutually exclusive occurrence in multi-factor models—where retained factors exhibit independent effects—suggests that that the race-ethnicity association with composition is closely related to a broader socioeconomic profile in both neonates and infants, and that this relationship is better captured by different factors at different ages.

### Maternal profiles of early life factors associated with microbiome composition

In addition to determining the individual factors contributing to gut microbiome composition, it is also important to assess whether combinations of these factors cluster to identify distinct profiles of mothers and whether such combinations synergistically explain additional variation in composition, beyond the individual factor effects alone. We therefore used a latent class analysis (LCA) to determine if there was evidence for different underlying profiles of mothers. We specifically focused on the earliest period of gut microbiome development captured in our study (the neonatal period), as our results show that these drivers shape composition in both neonates and infants (Fig. 3). The LCA results from the entire WHEALS cohort (n = 1,258 mothers; Supplementary Table S1) suggested that the three group maternal profile solution was the best fit to the data (bootstrap likelihood ratio p-value < 0.001).

The frequencies of the component factors for each of the three maternal profiles are presented in Table 1. Microbiome-associated maternal profile 1 (MMP1) was composed exclusively of African American mothers and had the lowest level of pet-keeping (11% had pets in the home at the neonatal visit). MMP2 had the least African American women (84% Caucasian/Other), and were most likely to be married at the pre-delivery visit (94%) and breastfeed at the neonatal visit (78% mixed or exclusive breastfeeding). MMP3 was the least frequent grouping, comprising 14% (n = 175) of mothers; notably, MMP3 was also the most racially diverse, with nearly equal percentages of African American (53%) and non-African American mothers (47%). MMP3 mothers were least likely to be married at pre-delivery (20%), least likely to breastfeed at the neonatal visit (3%), and most likely to report infant ETS exposure at the neonatal visit (87%).

As would be expected, there were compositional differences in the neonate gut microbiome between the three groups (unweighted UniFrac p-value < 0.001). Interestingly, when MMP grouping was included in the neonatal unweighted UniFrac multi-factor composition model, there was suggestive evidence that the groups explained an additional 1.7% of the total microbiome variation (p = 0.062), indicating that the MMPs capture additional contextual information between the factors that exceed their independent contributions. These results demonstrate the complexity and synergy of the combined effects of pregnancy, sociocultural, and environmental factors in distinguishing gut bacterial communities.

## Discussion

Our study has taken a comprehensive approach to the identification of pregnancy, sociocultural, and environmental factors related to early life gastrointestinal bacterial microbiota in a racially and socioeconomically diverse birth cohort. Unique among similar studies, we have not only applied single factor (i.e. univariate) analyses but also multi-factor (i.e. multivariate) approaches to identify parsimonious sets of factors influencing early life microbiome composition and identify maternal groupings that exhibit specific patterns of these factors. In doing so, we have introduced a novel approach that may be useful for capturing the joint effects of multiple exposures in early life and subsequent health and disease outcomes mediated by specific patterns of early life bacterial colonization.

The current study did not have longitudinal microbiome measures to assess the within-child impact of factors on changes in microbiome composition over time. However, the two age groups studied did allow for independent validation that 17 of the 19 individual factors associated with gut bacterial compositional differences in the neonatal subjects remained significantly associated in the infant subjects. The two factors that did not remain associated were ETS exposure during pregnancy and ETS exposure at the neonatal visit. However, in our infant multi-factor models, maternal smoking was identified as a significant factor associated with microbiome composition, suggesting that tobacco smoke in general is an important determinant of the developing gut microbiome composition across the first year of life.

Our findings were not only internally consistent, but they also largely agreed with previous studies. Breastfeeding, mode of delivery, gestational age, age of the child, number of previous pregnancies, and parity have all been previously associated with early life gut microbiome composition^8^,^10^,^13^,^20^,^21^. Additionally, we did not detect an effect of solid food introduction, consistent with the recent findings of Bäckhed *et al*.^7^, which suggested that cessation of breastfeeding rather than introduction of solid foods strongly influences developing microbial communities.

In contrast, we notably failed to detect compositional differences by antibiotic exposure both prenatally and in early life, which have previously been reported in the literature as important determinants of gut microbiome development^12^,^22^. However, our findings may be a reflection of the granularity of the definitions used (i.e. not classified by specific medication type or reason(s) for medication use), which were limited due to the low frequency of early life antibiotic use (3% among neonates; 22% among infants). Further, the definition of prenatal antibiotic use encompassed the entire pregnancy through date of delivery; future work in our and other cohorts is needed to determine if timing of antibiotic exposure during pregnancy is associated with offspring gut microbiome development.

A novel aspect of the present study is the representation of multiple race-ethnicities. In particular, we found that self-report of African American race-ethnicity is associated with gut microbiome compositional differences compared with non-African Americans in both neonates and infants. The constructed networks identified demographic factors that may be closely related. For example, household income, marital status, and maternal education were closely related to African American race-ethnicity in terms of commonly associated taxa. These factors are also significantly correlated with one another, and they may collectively be representative of an underlying sociocultural construct affecting gut microbiome composition. However, other unmeasured factors associated with African American race-ethnicity, including heritable genetic variation, cannot be ruled out as possibly contributing to this effect. Indeed, recent studies have demonstrated host germline genetic variation contributes to the differential abundance of certain taxa, suggesting that microbial composition may be partially heritable. Goodrich *et al*. recently demonstrated that *Christensenella*, a bacterial member of the gut microbiome, is heritable, with 40% of its variance in abundance attributable to additive genetic factors^23^. As African Americans are an admixed population composed of genetic ancestry from both Africa and Europe^24^, future studies should evaluate whether percent genome-wide African ancestry in African Americans is associated with early life gut bacterial composition to determine whether differences in ancestral genetic variation partially accounts for the effect of self-identified African American race-ethnicity.

Many of the differences in bacterial abundance by early life factors are consistent with previous studies. For example, we found that babies born via C-section had lower abundances of *Bacteroides* compared to babies born vaginally^7^,^9^,^25^. Additionally, we found that breastfeeding was associated with increased abundance of *Staphylococcus* taxa among neonates and *Bifidobacerium* and *Lactobacillus* taxa among infants, each of which have been associated with breastfeeding in previous studies^26^,^27^,^28^. In addition to these early life exposures that have been frequently examined in studies of infant gut microbiome composition, we also identified taxa-specific differences by several factors that have not been well characterized in terms of associations with infant gut microbiota. Many of these factors relate to socioeconomic status, which is an important determinant of human health^29^ and may indirectly impact early life gut microbiota through a variety of mechanisms, including environmental exposures (housing conditions, pollution, etc.), chronic stress, diet, and physical activity. These findings as well as the potential context-dependent effects suggested by the MMP groups will need to be validated by additional studies of socioeconomically and racially diverse populations, which are currently under-represented in the microbiome literature.

Our multi-factor approach also allowed us to identify the presence of indoor pets as significantly and independently associated with gut microbiome composition in neonates. We and others have shown that exposure to pets in early life protects children against the development of allergic disease^30^ and that indoor pets significantly alter the diversity of the microbiome of the home, as measured in dust samples^31^,^32^,^33^. Further, a recent publication from our group demonstrated that murine exposure to house dust from dog-keeping homes affected the gut microbiome^34^. Taken together, these findings support the hypothesis that the associations between pet exposure and allergic outcomes may be mediated by the effect of gut microbial composition changes due to pet exposure in the first few months following birth.

In addition to our multi-factor composition models, the latent class analysis identified maternal profiles associated with early life gut microbiome composition that would not have been hypothesized based solely on pairwise correlations between the factors. In particular, MMP3 mothers reported the highest rates of ETS exposure (87%) and the lowest rates of both breastfeeding (3%) and being married at delivery (20%). This non-racially disparate group reflects a susceptibility profile with potentially detrimental health effects that may be mediated by early life gut microbiome composition. Further studies are needed to understand potential associations between MMP groups and early childhood conditions, such as allergic disease.

In aggregate, the factors in the age-group specific unweighted UniFrac multi-factor models explained 12–14% of the variability in the microbiome. While not a majority, there are numerous explanations for these modest effects. First, there are limitations to the granularity of information that can be derived from questionnaire data. For example, the assessment of breastfeeding fails to capture the complexity of breast milk content (oligosaccharides, lipids, metabolites, cytokines, etc.), which is known to be highly variable between mothers^35^. These bioactive components could be profiled by modern molecular techniques to provide a finer-grained assessment of breast milk content that may explain more of the variability in gut microbiome composition. Further, breastfeeding status was coarsely classified as exclusive, any, or none. While this is standard in the literature, it does not recognize the heterogeneity that exists within each of these categories, such as the other dietary factors to which the baby is exposed. Food frequency questionnaires are an epidemiological tool that could be used for both the mother and her baby to better capture this heterogeneity. Finally, it is known that interpersonal microbiome variability is high, with a majority of rare taxa present^36^. As a result, the proportion of gut compositional variability that can be explained by common exposures is effectively limited.

A growing body of literature has identified gut microbiome perturbations as associated with a range of diseases^37^. Our findings have particular relevance to epidemiologic studies on the developmental origins of diseases, where mounting evidence suggests that alterations in early life bacterial composition are related to subsequent disease development^2^,^3^,^4^,^5^,^6^. A broad understanding of the environmental and sociocultural factors that may influence the early life gut microbiome is necessary for the proper design and analysis of such studies. Inappropriate control for such factors in either the design (e.g. matching) or analysis (e.g. adjustment as a covariate) may produce misleading results, as the portion of microbiome variation explained by these factors may lie directly on the causal pathway between exposure and outcome. Further, our study has identified that complex interactions between these factors are associated with microbiome composition alteration. While these suggestive findings need to be confirmed by additional studies, they indicate that not accounting for such context-dependent effects may lead to inconsistent results when the microbiome is a complete or partial mediator of the exposure-disease relationship.

In summary, our approach to characterizing pregnancy, sociocultural, and environmental factors associated with gut microbiome moves this field of study beyond single factor analyses to provide multi-factor insights into compositional differences between children in early life. Our study also has identified African American race-ethnicity as having important independent and context-dependent effects on early life gut bacterial composition and underscores the need for more studies of under-represented minorities^38^. This is especially true for the study of disease outcomes that are racially disparate in terms of risk, and where the microbiome is hypothesized to have a causal effect.

## Methods

### Study population

Analyses were performed on data and samples collected from the WHEALS birth cohort based in and around Detroit, Michigan, USA. WHEALS recruited pregnant women with due dates from September 2003 through December 2007, and who were seeing a Henry Ford Health System (HFHS) practitioner at one of five clinics to establish an unselected birth cohort. All women were in their second trimester or later, were aged 21–49 years, and were living in a predefined contiguous geographic area in Wayne and Oakland counties that included the city of Detroit as well as the suburban areas immediately surrounding the city. Post-partum interviewer-administered questionnaires and in-person evaluations were completed periodically, including survey and home visits targeted for ages 1 and 6 months. Delivery records for WHEALS women were abstracted to obtain delivery type (vaginal or C-section), birth weight, and gestational age at delivery. All participants provided written, informed consent, and study protocols were approved by the Institutional Review Board at HFHS. Further, the study was performed in accordance with the protocol guidelines approved by the Institutional Review Board at HFHS.

The WHEALS cohort included 1,258 babies; 255 were dropped for non-compliance. Of the remaining 1,003 eligible, 763 children (76.1%) either completed a 2 year follow-up visit in the clinic or had blood drawn for measurement of immunoglobulin E (primary outcome of parent study). Of these children, we determined those who had an available stored paired house dust and stool sample collected at the same 6-month, or if not available, 1-month visit. Stool samples from 308 children underwent microbial sequencing; of these, 298 (n = 130 from the 1-month visit [age range 0.5–4.6 months], referred to as “neonates”, and n = 168 from the 6-month visit [age range 5.6–10.6 months], referred to as “infants”) were successfully sequenced and comprise the analytic sample (Supplementary Figure S1).

We assessed numerous population characteristics of the selected sample versus the entire birth cohort and found few statistically significant differences between the two (Supplementary Table S1). Of particular note, race (61% African American), marital status (63.6% married), mode of delivery (38.3% C-section), and environmental tobacco smoke (ETS) exposure (26.0% during pregnancy) were similar (all p-values > 0.20). Report of ever breastfed was also similar between the analytic sample and parent sample (78.1% vs. 77.9%, respectively; p = 0.95). However, rate of current breastfeeding at the 1-month visit was higher in the analytic sample (56.1% vs 48.6%, respectively; p = 0.028). A larger percentage of the analytic sample came from households with higher income (30.8% vs. 19.8% reported greater than $80,000 household income, respectively; p < 0.001). We also evaluated differences between the neonates (n = 130) and infants (n = 168) used in the current analysis (Supplementary Table S1). The two groups were similar, with the following exceptions (all p < 0.05): infants tended to have fewer siblings, were more likely to be delivered via C-section, less likely to be born in the winter, less likely to have detectable Der f in the household, and more likely to reside in a home built before 1950.

### Environmental and lifestyle measurements

Unless described differently, the pre- and post-natal interviews with the mother were the sources for most of the factors included in this manuscript. A complete description of each of the factors used is presented in Supplementary Table S2. The child’s mode of delivery, birth weight, and gestational age, along with the mother’s medication (antibiotic/antifungal) use was abstracted from maternal and delivery medical records. Mother’s BMI during the first trimester was also abstracted from medical records. Though maternal BMI was not typically available prior to pregnancy, BMI in the first trimester is a reasonable marker of pre-pregnancy BMI^39^. Early life medication use was abstracted from infant medical records. Gender- and gestational-age adjusted birthweight z-scores were calculated using the United States population as a ref. 40.

Dust samples were collected and analyzed using methods we have previously published^41^. Samples were collected from five locations within the residence (mother and child’s bedroom floors and mattresses along with the most used common area, which was typically the living room floor) at the 1- and 6-month home visits. All samples were analyzed for endotoxin, while the specific allergens of cat (Fel d 1), dog (Can f 1), and dust mite allergen (Der f) were only measured in the dust from the child’s bedroom floor. For these analyses, as done previously^41^, pet-keeping was defined as having a cat or dog or both indoors at least 1 hour of the day.

### Stool samples

Infant stool was collected by field staff during home visits targeting 1 and/or 6 months of age. Stool was placed into cryovials, transported to the laboratory and stored at −80 °C. DNA was extracted from stool samples using a modified cetyltrimethylammonium bromide (CTAB) buffer based protocol^42^. Briefly, modified CTAB extraction buffer (0.5 ml) were added to 25 mg of stool in a 2 ml Lysing Matrix E tube (MP Biomedicals, Santa Ana, CA) prior to incubation at 65 °C for 15 min. After samples were bead-beaten at 5.5 m s^−1^ for 30 sec in a Fastprep-24 (MP Biomedicals, Santa Ana, CA), 0.5 ml of phenol:chloroform:isoamyl alcohol (25:24:1) was added. Samples were then centrifuged (16,000 × g, 5 min) and the resulting supernatant was transferred to 2 ml heavy phase-lock gel tube (5 Prime, Gaithersburg, MD) to which chloroform (v/v) was added. Following a second centrifugation (12,000 × g, 5 min), the supernatants were placed in fresh 1.5 ml tubes where 1 μl of linear acrylamide and PEG/NaCl (2v/v) were added. Following a 2 h incubation at room temperature, samples were then centrifuged (16,000 × g, 10 min), washed with ice cold 70% EtOH and resuspended in 300 μl of 10 mM Tris-Cl, pH 8.5.

#### PCR conditions and library preparation for bacterial sequencing

Primer pair F515 (5′-GTGCCAGCMGCCGCGGTAA-3′) and R806 (5′-TAATCTWTGGGVHCATCAGG -3′), which included the Illumina flowcell adapter sequence and pad region^43^ were used to amplify the V4 region of the 16S rRNA gene^44^. Each reverse primer encoded a 12-base error correction Golay barcode unique for individual samples^44^. PCR reactions were performed in triplicate using 0.025 U Takara Hot Start ExTaq (Takara Mirus Bio Inc, Madison, WI), 1X Takara buffer with MgCl_2_, 0.4 pmol μl^−1^ of F515 and R806 primers, 0.56 mg ml^−1^ of bovine serum albumin (BSA; Roche Applied Science, Indianapolis, IN), 200 μM of dNTPs, and 10 ng of gDNA in 25 μl reactions. Thermocylcer conditions were as followed: initial denaturation (98 °C for 2 min) followed by 30 cycles of 98 °C (20 sec), annealing at 50 °C (30 sec), extension at 72 °C (45 sec) and a final extension at 72 °C for 10 min. Agarose e-gels (2% TBE; Life Technologies, Grand Island, NY) were used to verify successful amplification of pooled PCR amplicons, followed by purification using AMPure SPRI beads (Beckman Coulter, Brea, CA). Quality of the amplicons was verified with the Bioanalyzer DNA 1000 Kit (Agilent, Santa Clara, CA) and quantification by done using the Qubit 2.0 Fluorometer and the dsDNA HS Assay Kit (Life Technologies, Grand Island, NY). Libraries were made by pooling samples in equal moles at concentrations of 50 ng. Libraries then denatured and diluted to 2 nM, and 5 pM were loaded onto the Illumina MiSeq cartridge with 15% (v/v) of denatured 12.5 pM PhiX spike-in control.

#### Sequence data processing and quality control

Paired-end sequences were assembled using FLASH v 1.2.7^45^, de-multiplexed by barcode, and low quality reads (Q-score < 30) were discarded in QIIME 1.8^46^. Reads were truncated if three consecutive bases were < Q30, and the resulting read retained in the data set only if it was at least 75% of the original length. UCHIME^47^ was used to check for chimeras, which were filtered from the dataset prior to operational taxonomic unit (OTU) picking at 97% sequence identification using UCLUST^48^ against the Greengenes database version 13_5^49^; sequence reads that failed to cluster with a reference sequence were clustered *de novo*. Sequences were then aligned using PyNAST^50^, and taxonomy was assigned using the RDP classifier^51^ and Greengenes reference database version 13_5^49^. FastTree^52^ was then used to build a phylogenetic tree.

As rarefying the data once may result in an unrepresentative and outlying sample, particularly when many rare taxa are present, read depths were rarefied multiple times and the most “representative” rarefied dataset was selected. This representative approach to rarefying was defined for each subject as follows: for 100 subject-specific rarefied OTU vectors, the most representative one is selected, defined as the one that is the minimum average Euclidean distance from itself to all other OTU vectors, the idea being that upon repeated sampling, extreme subsamples are avoided by choosing one central to all others. Hence, the resulting representative rarefied OTU table was a concatenation of various subject-specific subsamples. The resulting OTU count table was representative-rarefied to the minimum depth of 202,367 total sequences per sample, and was the basis for all subsequent analyses involving the microbiome. As RDP classification resolves the sequences at different levels of taxonomy, we use the commonly accepted term “taxa” in the Results and Discussion rather than the technical term “OTU” for readability.

### Statistical analysis

Except where otherwise noted, all analyses were performed in R version 3.2.1^53^. Gross community measures of bacterial richness (number of unique OTUs present), Pielou’s evenness (relative distribution of OTUs in a community), and Faith’s phylogenetic diversity were estimated using QIIME and the R vegan package^54^, with tests of association between these measures and baseline characteristics conducted using Wilcoxon Rank Sum/Kruskal-Wallis tests (categorical variables) or Spearman’s correlations (continuous variables). As implemented using the adonis function in the R vegan package, permutational multivariate analysis of variance (PERMANOVA)^18^ was used to assess the relationship of baseline characteristics with microbiome composition, using unweighted and weighted UniFrac metrics^55^. We used both weighted and unweighted UniFrac metrics to capture different aspects of bacterial community composition as they relate to environmental and sociocultural factors, with the weighted version detecting shifts in the taxa relative abundances and the unweighted capturing the contribution of rarer taxa to these relationships^56^. In each univariate PERMANOVA model, 10,000 Monte Carlo permutations were utilized.

To determine the specific OTUs driving compositional differences, tests of differential OTU abundance were performed using zero-inflated negative binomial regression; in cases where zero-inflated models failed to converge, the standard negative binomial was implemented as a secondary modeling strategy. To avoid testing overly sparse taxa, only OTUs with at least 5 total sequence reads across all samples were tested. Some modifications had to be made for OTU testing on multi-category predictors due to sparsity. Specifically, income was dichotomized to ≥$80,000, education was dichotomized to ≥bachelor’s degree, marital status was tested using an indicator for married, maternal race and baby race was tested as African American vs. other, and indoor pets was tested using an indicator for any indoor pets. Statistical significance was assessed after accounting for multiple OTU testing using the Benjamini & Hochberg false discovery rate (FDR) adjustment, with FDR adjusted p-values < 0.05 considered significant^57^.

#### Multi-factor models of infant gut microbiota composition

In order to determine the early life factors independently associated with microbiome composition at both 1 and 6 months, we performed backward elimination on PERMANOVA models, where predictors with type III p-values < 0.05 were retained in the model (1,000 permutations). Because PERMANOVA is not equipped to handle missing values and large multivariate models would result in sparse sample sizes, the data was first imputed such that missing values of continuous predictors were replaced with the mean, and missing values of categorical predictors were made into a missing category (contrary to single-factor tests in which missing values were omitted). Given the low rates of missingness across the factors (all <8% except for household income and baby antibiotic use; missing counts and percentages for all of the factors are included in Supplementary Table S2), the bias due to missingness is expected to be minimal^58^. Additionally, because several predictors had multiple measurements taken at different time points (pregnancy, 1 month, and 6 months), we removed redundant variables from the model selection procedure to avoid multicollinearity issues. Specifically, we calculated the variance inflation factors (VIFs) between these multiple measurements and used the following rule: if two measurements were available with VIF > 2.5, the closest measurement to stool collection time was retained; otherwise, both measurements were retained. If three measurements were available with at least one VIF > 2.5, the two measurements furthest apart were retained, and the pairwise VIF was recalculated to potentially capture a wider range of exposure. If the remaining pairwise VIF > 2.5, then only the closest measurement to stool collection time was retained. Additionally, all final multivariate models were examined for large VIFs; the largest VIF detected was 2.5, indicating stability in these models.

#### Networks of factors demonstrating shared impact on gut microbial composition

For both the 1- and 6-month study visit, network plots were created to demonstrate the relationship between the factors based on shared microbial composition impact, which were constructed using the ggnet2 function of the GGally package in R^59^. Factors were considered the nodes and similarity in terms of association with specific taxa were considered the edges between the nodes. Factors were only included in the network plots if either significant single- or multi-factor compositional differences were found. In order to define compositional similarity for each pair of factors, we calculated the percentage of overlapping significant OTUs among all significant OTUs between the two factors. Intuitively, this provides a measure of how similar the specific OTUs associated with two factors were. A threshold of at least the 80^th^ percentile in these proportions was used to define the network adjacency matrix, meaning 20% of all possible between-variable connections were made in each network. For the neonatal network, this meant that two factors had to have at least 15% overlap in shared associated taxa to be connected. In the infant network, at least 12% overlap had to be achieved. To help evaluate the extent to which similarity in composition is due to similarity between the factors, we also calculated between-factor correlations using either Pearson correlation (when both variables were continuous), point-biserial correlation (when there was one continuous and one binary variable), or the Phi coefficient (when both variables were binary). Connections in the network plots were highlighted to indicate those with significant (p < 0.05) correlations; correlation matrix heatmaps of all factors included in the network plots (for both neonates and infants) were also constructed.

#### Determination of microbiome-associated maternal profiles

Latent class analysis (LCA) is a statistical method for identifying underlying groups of similar individuals. Briefly, LCA tests the hypothesis that subjects come from a heterogeneous population, i.e. that there is more than one underlying (latent) homogeneous sub-population that the subjects have been drawn from. The size and number of the underlying groups are unknown *a priori* and thus are data driven. By employing LCA solely on the maternal variables included in the final neonatal multi-factor PERMANOVA models, we are able to test for the existence of *microbiome-associated maternal profiles* (MMPs).

LCA was performed using a set of six maternal categorical variables (maternal race, marital status, mode of delivery, breastfeeding practices at 1-month interview (exclusive, current but not exclusive and none), pet(s) at 1-month, and ETS at the 1-month interview). Age of stool was excluded as a non-maternal factor. Models with increasing number of groups (n = 1 to 5) were tested and compared for goodness-of-fit. The three-group solution was selected based on a statistically significant bootstrapped likelihood ratio test (indicated that 3 profiles were necessary (p < 0.001), but 4 were not (p = 0.27)) and the minimum sample size adjusted Bayesian Information Criteria (BIC). Analyses were performed using PROC LCA and %LCABootstrap in SAS 9.4^60^.

LCA was performed on the entire WHEALS cohort (n = 1,258 Mothers). We tested for measurement invariance between the 130 that comprised the 1-month visit sample dataset and the remaining 1,128. No evidence was found to reject the null hypotheses that the underlying groups were different between those with and without microbiome data at the 1 month visit (p = 0.83), suggesting a valid result that is not unique to those subjects with neonatal microbiome data. This was consistent with our overall findings of only minimal demographic differences between cohort members included and excluded from MAAP (Supplementary Table S1).

Each subject was assigned to the MMP group with the highest posterior probability to provide descriptive characteristics to assist in interpretations and to test for the additional percent variation explained by MMP groups in the neonatal multi-factor adonis model. The mean maximum posterior probabilities for the 130 group were 0.74, 0.94 and 0.82, respectively for groups 1–3, indicating low classification error.

### Data and Materials Availability

16S sequence reads were deposited to the European Bioinformatics Institute (EBI) with accession number PRJEB13896 (http://www.ebi.ac.uk/ena/data/view/PRJEB13896).

## Additional Information

**How to cite this article**: Levin, A. M. *et al*. Joint effects of pregnancy, sociocultural, and environmental factors on early life gut microbiome structure and diversity. *Sci. Rep.* **6**, 31775; doi: 10.1038/srep31775 (2016).

## Supplementary Material

Supplementary Information

Supplementary Table S5

## Figures and Tables

**Table 1:** Description of microbiome-associated maternal profiles (MMPs) in the WHEALS cohort (N = 1,258).

Factor	Level	MMP1 N = 609 (48%)	MMP2 N = 474 (38%)	MMP3 N = 175 (14%)	p-values*			
		N (Column %)			Overall	MMP1 vs. MMP2	MMP1 vs. MMP3	MMP2 vs. MMP3
Maternal Race-Ethnicity	African American	609 (100)	77 (16.2)	92 (52.6)	<0.001	<0.001	<0.001	<0.001
	Caucasian/Other	0 (0)	397 (83.8)	83 (47.4)				
Married	Yes	293 (48.1)	445 (93.9)	35 (20)	<0.001	<0.001	<0.001	<0.001
	No	316 (51.9)	29 (6.1)	140 (80)				
Mode of Delivery	Vaginal	364 (60.1)	315 (66.7)	105 (61)	0.071	0.024	0.82	0.18
	C-section	242 (39.9)	157 (33.3)	67 (39)				
Breastfeeding Practices - NSV	None	266 (59)	90 (21.5)	145 (96.7)	<0.001	<0.001	<0.001	<0.001
	Mixed	171 (37.9)	205 (49)	5 (3.3)				
	Exclusive	14 (3.1)	123 (29.4)	0 (0)				
Indoor Pet(s) - NSV	Yes	51 (11.3)	204 (48.8)	66 (44)	<0.001	<0.001	<0.001	0.31
	No	400 (88.7)	214 (51.2)	84 (56)				
ETS - NSV	Yes	51 (11.3)	18 (4.3)	130 (86.7)	<0.001	<0.001	<0.001	<0.001
	No	400 (88.7)	401 (95.7)	20 (13.3)				

Abbreviations: MMP, microbiome-associated maternal profiles; NSV, neonatal study visit; ETS, environmental tobacco smoke.

*Chi-square p-values.
