# Supplementary material for: Joint effects of pregnancy, sociocultural, and environmental factors on early life gut microbiome structure and diversity
Source: Sci Rep. 2016 Aug 25;6:31775. doi: 10.1038/srep31775 (PMC4997337; doi:10.1038/srep31775)
Supplement: Supplementary Information [file srep31775-s1.pdf]

Joint effects of pregnancy, sociocultural, and environmental factors on early life gut microbiome structure and diversity.

Albert M. Levin[1,2*], Alexandra R. Sitarik[1*], Suzanne L. Havstad[1], Kei E. Fujimura[3], Ganesa Wegienka[1], Andrea E. Cassidy-Bushrow[1], Haejin Kim[4], Edward M. Zoratti[4], Nicholas W. Lukacs[5], Homer A. Boushey[6], Dennis R. Ownby[7], Susan V. Lynch[3†], Christine C. Johnson[1†]

[1] Department of Public Health Sciences, Henry Ford Health System, Detroit, MI, 48202;
[2] Center for Bioinformatics, Henry Ford Health System, Detroit, MI, 48202;
[3] Division of Gastroenterology, Department of Medicine, University of California, San Francisco, CA, 94143;
[4] Division of Allergy and Clinical Immunology, Department of Medicine, Henry Ford Health System, Detroit, MI, 48202;
[5] Department of Pathology, University of Michigan, Ann Arbor, MI, 48109;
[6] Division of Pulmonary and Critical Care Medicine, Department of Medicine, University of California, San Francisco, CA, 94143;
[7] Department of Pediatrics, Georgia Regents University, Augusta, GA, 30912

* These authors contributed equally to this work
[†] These authors share co-senior authorship for this work

Supplementary Information

Supplementary Figures

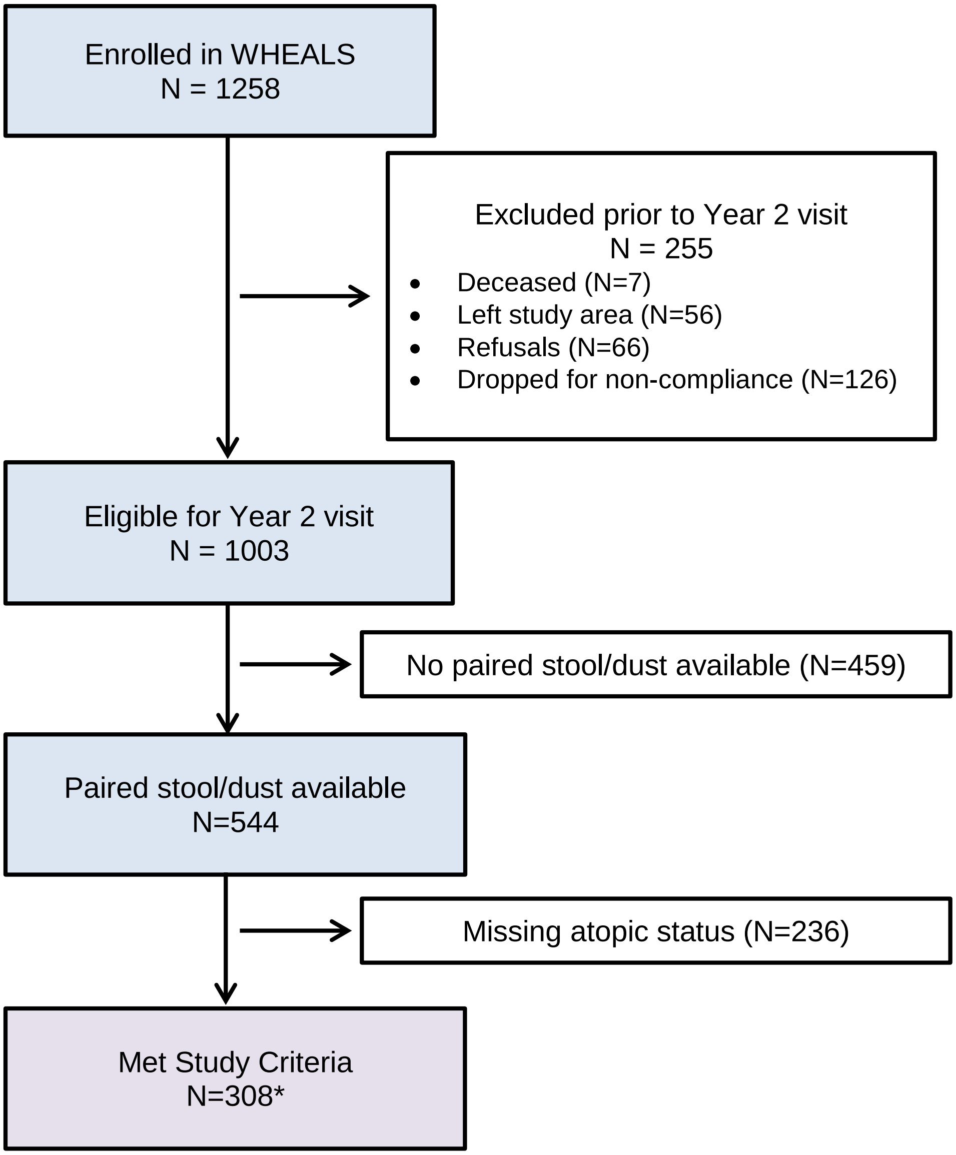

*N=298 stool samples with sufficient DNA for sequencing

Supplementary Figure S1: Inclusion/exclusion criteria and number of participants meeting study criteria. Of the 308 participants that met study criteria and had stool samples that underwent microbial analyses, 298 (n=130 from the 1-month visit and n=168 from the 6-month visit) were successfully sequenced and comprise the (statistical) analytical sample.

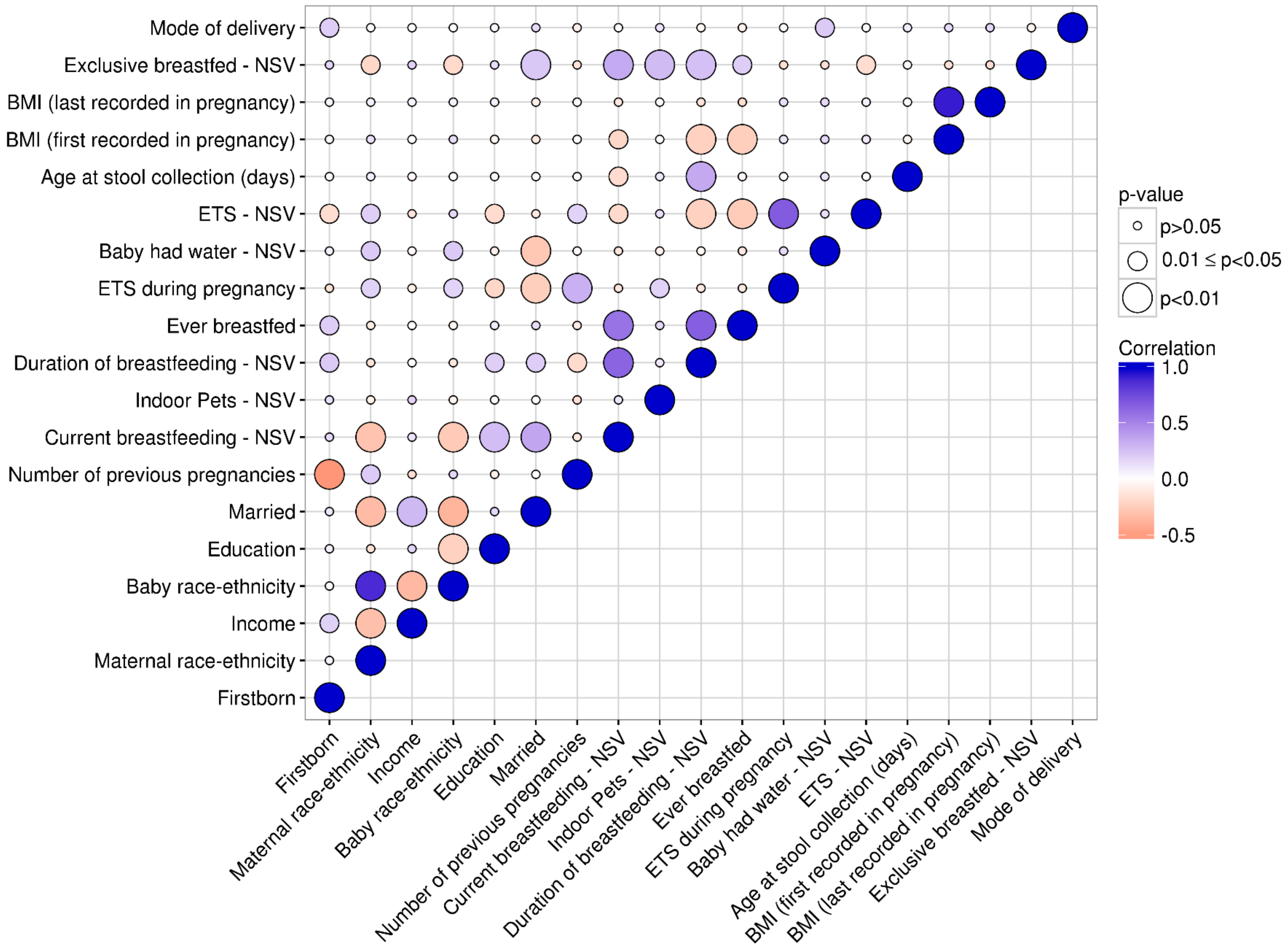

Supplementary Figure S2: Between-factor correlations among neonates. The heatmap includes factors associated with compositional differences in the neonatal gut microbiome in either single or multi-factor models. Correlations are Pearson, Point Biserial, or Phi Coefficients, as appropriate. Abbreviations: BMI, body mass index; ETS, environmental tobacco smoke; NSV, neonatal study visit at 1-month of age.

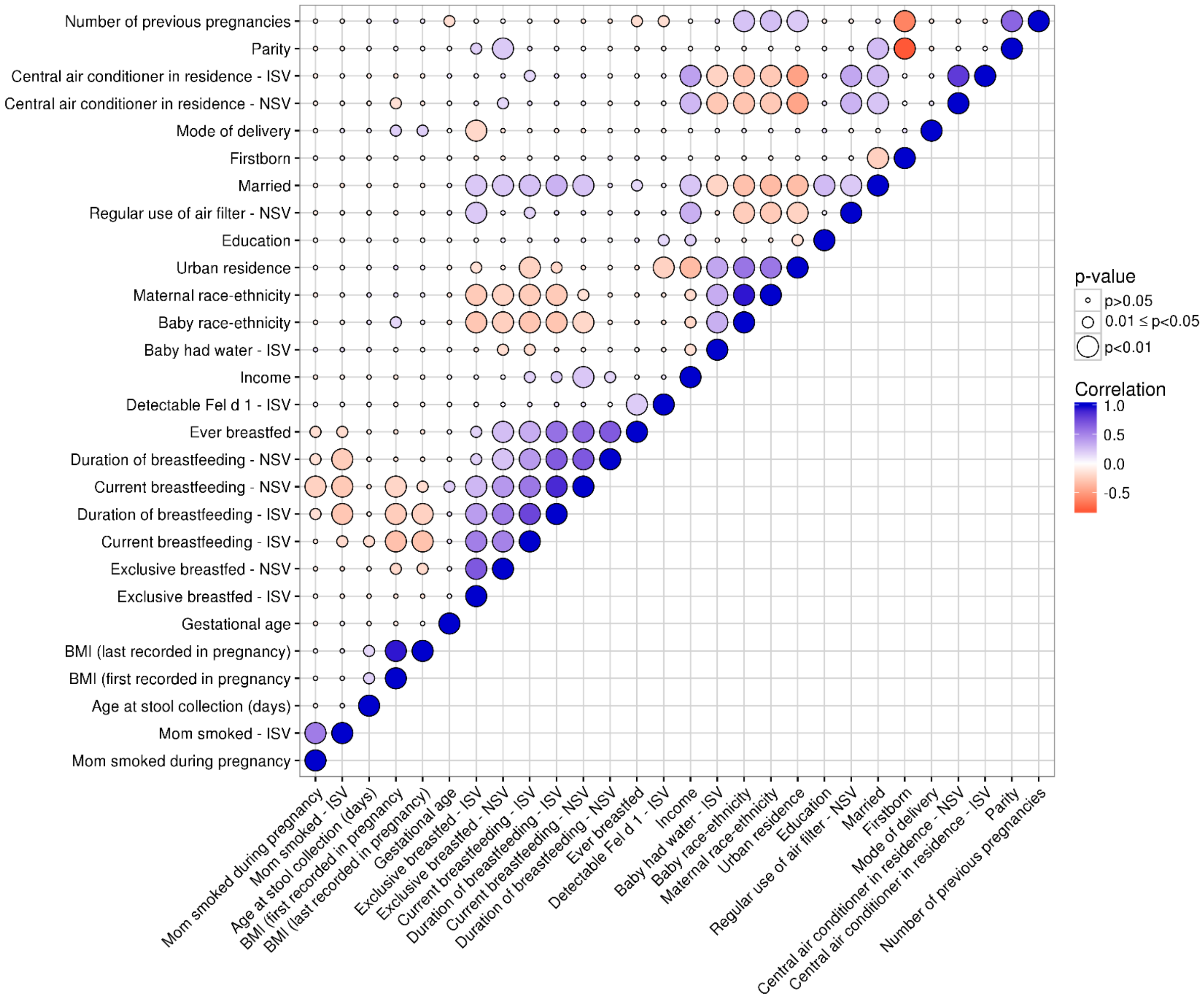

Supplementary Figure S3: Between-factor correlations among infants. The heatmap includes factors associated with compositional differences in the infant gut microbiome in either single or multi-factor models. Correlations are Pearson, Point Biserial, or Phi Coefficients, as appropriate. Abbreviations: BMI, body mass index; NSV, neonatal study visit at 1-month of age; ISV, infant study visit at 6-months of age.

Supplementary Tables

Supplementary Table S1: Comparison of characteristics between WHEALS subjects included and excluded from the MAAP study.

	MAAP Included			MAAP Excluded	WHEALS		
	NSV (N=130) N (%)	ISV (N=168) N (%)	Total (N=308) N (%)	Total (N=950) N (%)	Total (N=1258) N (%)	p-value*	Missing N
Demographics							
**Maternal age‡**	29.8 (5.2)	29.8 (5.3)	29.8 (5.2)	29.5 (5.3)	29.6 (5.2)	0.41	
**Paternal age‡**	32.1 (6.9)	33.4 (7.1)	32.7 (7.0)	32.1 (6.7)	32.3 (6.8)	0.18	*92*
**Maternal race-ethnicity**						0.28	
Caucasian	31 (23.9%)	48 (28.6%)	82 (26.6%)	208 (21.9%)	290 (23.1%)		
African American	82 (63.1%)	100 (59.5%)	188 (61.0%)	590 (62.1%)	778 (61.8%)		
Hispanic	8 (6.1%)	6 (3.6%)	15 (4.9%)	63 (6.6%)	78 (6.2%)		
Arab American	5 (3.8%)	9 (5.3%)	14 (4.6%)	45 (4.7%)	59 (4.7%)		
Other	4 (3.1%)	5 (3.0%)	9 (2.9%)	44 (4.6%)	53 (4.2%)		
**Married**	75 (57.7%)	114 (67.9%)	196 (63.6%)	577 (60.7%)	773 (61.4%)	0.36	
**Urban residence**	71 (54.6%)	83 (49.4%)	159 (51.6%)	544 (57.3%)	703 (55.9%)	0.083	
**Household income**						<0.001	
<$40,000	45 (34.6%)	50 (29.8%)	99 (32.1%)	378 (39.8%)	477 (37.9%)		
$40,000-$80,000	31 (23.9%)	40 (23.8%)	73 (23.7%)	274 (28.8%)	347 (27.6%)		
>$80,000	39 (30.0%)	54 (32.1%)	95 (30.8%)	188 (19.8%)	283 (22.5%)		
Refused/Did not answer	15 (11.5%)	24 (14.3%)	41 (13.3%)	110 (11.6%)	151 (12.0%)		
**Maternal education**						0.18	
Less than high school	5 (3.8%)	7 (4.2%)	13 (4.2%)	61 (6.4%)	74 (5.9%)		
High school	23 (17.7%)	21 (12.5%)	50 (16.2%)	178 (18.7%)	228 (18.1%)		
More than high school	102 (78.5%)	140 (83.3%)	245 (79.6%)	711 (74.9%)	956 (76.0%)		
Pregnancy and Delivery							
**Parity‡**	1.17 (1.24)	0.79 (0.89)†	0.96 (1.07)	1.19 (1.28)	1.14 (1.23)	0.002	*2*
**C-section delivery**	41 (31.5%)	74 (44.0%)†	118 (38.3%)	348 (36.9%)	466 (37.3%)	0.67	*8*
**Firstborn**	45 (34.6%)	76 (45.2%)	124 (40.3%)	336 (35.4%)	460 (36.6%)	0.12	*1*
**Number of previous pregnancies‡**	1.94 (1.84)	1.62 (1.48)	1.78 (1.68)	2.07 (1.87)	2.00 (1.83)	0.011	*2*
**Male baby**	71 (54.6%)	86 (51.2%)	164 (53.2%)	460 (48.5%)	624 (49.6%)	0.15	*1*
**Maternal antibiotic use during pregnancy**	68 (54.4%)	85 (54.1%)	158 (54.3%)	250 (55.2%)	408 (54.8%)	0.81	*514*
**Maternal antifungal use during pregnancy**	21 (16.8%)	36 (22.9%)	58 (19.9%)	82 (18.1%)	140 (18.8%)	0.53	*514*
**BMI (first recorded during pregnancy)‡**	30.2 (7.4)	30.6 (8.4)	30.4 (8.1)	30.4 (8.1)	30.4 (8.1)	0.95	*463*
**BMI (last recorded during pregnancy)‡**	34.7 (7.4)	35.2 (7.9)	34.9 (7.7)	35.0 (7.8)	34.9 (7.7)	0.97	*463*
**Birth weight z-score‡**	-0.05 (0.99)	-0.11 (1.00)	-0.09 (1.00)	-0.13 (0.99)	-0.12 (0.99)	0.61	*86*
**Gestational age‡**	38.9 (1.5)	38.7 (1.8)	38.8 (1.7)	38.7 (1.8)	38.7 (1.8)	0.56	*26*

	MAAP Included			MAAP Excluded	WHEALS		
	NSV (N=130) N (%)	ISV (N=168) N (%)	Total (N=308) N (%)	Total (N=950) N (%)	Total (N=1258) N (%)	p-value*	Missing N
**Season of birth**						0.78	
Winter	36 (27.7%)	21 (12.5%)†	57 (18.5%)	200 (21.0%)	257 (20.4%)		
Spring	29 (22.3%)	39 (23.2%)	74 (24.0%)	211 (22.2%)	285 (22.7%)		
Summer	31 (23.8%)	52 (31.0%)	85 (27.6%)	260 (27.4%)	345 (27.4%)		
Fall	34 (26.2%)	56 (33.3%)	92 (29.9%)	279 (29.4%)	371 (29.5%)		
**Baby race-ethnicity**						0.73	
Caucasian	25 (19.2%)	40 (23.8%)	67 (21.8%)	200 (21.1%)	267 (21.2%)		
African American	83 (63.9%)	102 (60.7%)	191 (62.0%)	595 (62.6%)	786 (62.5%)		
Hispanic	11 (8.5%)	9 (5.4%)	22 (7.1%)	57 (6.0%)	79 (6.3%)		
Arab American	5 (3.8%)	12 (7.1%)	17 (5.5%)	48 (5.0%)	65 (5.2%)		
Other	6 (4.6%)	5 (3.0%)	11 (3.6%)	50 (5.3%)	61 (4.8%)		
Feeding practices – NSV							
**Ever breastfed**	101 (77.7%)	129 (77.7%)	230 (78.1%)	579 (77.9%)	818 (78.0%)	0.95	*209*
**Exclusively breastfed**	16 (12.3%)	30 (18.6%)	49 (16.3%)	88 (12.3%)	137 (13.4%)	0.086	*239*
**Currently breastfed**	70 (53.9%)	91 (56.5%)	169 (56.1%)	349 (48.6%)	518 (50.8%)	0.028	*239*
**Duration of breastfeeding (in days)‡**	24.0 (19.4)	22.1 (17.8)	23.6 (19.2)	24.4 (22.1)	24.1 (21.3)	0.58	*241*
**Baby had water**	33 (25.4%)	29 (18.0%)	65 (21.6%)	180 (25.1%)	245 (24.1%)	0.23	*240*
**Baby had antibiotic(s)**	3 (2.6%)	5 (5.6%)	8 (3.8%)	10 (3.9%)	18 (3.8%)	0.96	*789*
Feeding practices – ISV							
**Exclusively breastfed**	9 (7.0%)	16 (9.5%)	26 (8.5%)	36 (4.8%)	62 (5.8%)	0.018	*195*
**Currently breastfed**	28 (23.5%)	46 (27.4%)	78 (26.3%)	156 (21.6%)	234 (23.0%)	0.11	*240*
**Duration of breastfeeding (in months)‡**	2.6 (2.6)	3.1 (2.9)	2.9 (2.8)	2.5 (2.8)	2.6 (2.8)	0.043	*309*
**Baby had water**	75 (68.8%)	115 (68.4%)	197 (68.6%)	427 (68.0%)	624 (68.2%)	0.85	*343*
**Early solid food (< 4 months of age)**	47 (36.2%)	71 (42.3%)	122 (39.6%)	287 (39.1%)	409 (39.2%)	0.87	*215*
Environment – during pregnancy							
**Indoor pet(s)**	49 (37.7%)	62 (36.9%)	116 (37.7%)	322 (33.9%)	438 (34.8%)	0.23	
**ETS**	33 (25.4%)	43 (25.6%)	80 (26.0%)	267 (28.1%)	347 (27.6%)	0.47	
Environment – NSV							
**Indoor pet(s)**	44 (33.9%)	50 (31.1%)	99 (32.9%)	222 (30.9%)	321 (31.5%)	0.54	*239*
**ETS**	24 (18.5%)	26 (16.2%)	54 (17.9%)	145 (20.2%)	199 (19.5%)	0.41	*238*
**Mom smoked**	14 (10.8%)	12 (7.5%)	27 (9.0%)	79 (11.0%)	106 (40.4%)	0.33	*239*
**Endotoxin - Mother's mattress§**	3.2 (1.3-6.7)	3.5 (1.3-8.1)	3.2 (1.3–7.2)	3.8 (1.5–8.9)	3.5 (1.5–8.5)	0.17	*307*
**Endotoxin - Mother's bedroom floor§**	14.9 (4.7-39.4)	13.7 (4.7-29.3)	14.2 (4.7–35.5)	14.6 (5.1–34.0)	14.4 (5.0–34.3)	0.62	*316*
**Endotoxin - Baby's mattress§**	2.8 (1.2-6.1)	2.4 (0.9-5.5)	2.7 (1.1–5.5)	2.6 (1.0–6.0)	2.6 (1.0–5.8)	0.83	*276*
**Endotoxin - Baby's bedroom floor§**	12.7 (4.1-38.4)	14.2 (4.9-32.4)	13.9 (4.4–35.9)	13.5 (4.9–33.0)	13.7 (4.8–33.6)	0.77	*291*
**Endotoxin - living room floor§**	20.6 (9.4-49.9)	22.0 (6.5-43.4)	20.7 (7.9–45.2)	21.0 (8.8–46.2)	20.9 (8.6–46.0)	0.99	*295*

	MAAP Included			MAAP Excluded	WHEALS		
	NSV (N=130) N (%)	ISV (N=168) N (%)	Total (N=308) N (%)	Total (N=950) N (%)	Total (N=1258) N (%)	p-value*	Missing N
**Detectable Can f 1**	79 (62.7%)	94 (60.3%)	180 (61.6%)	415 (59.0%)	595 (59.8%)	0.44	*263*
**Detectable Fel d 1**	83 (65.4%)	117 (76.0%)	209 (71.8%)	559 (79.5%)	768 (77.3%)	0.008	*264*
**Detectable Der f 1**	40 (31.5%)	58 (37.4%)	101 (34.6%)	271 (38.7%)	372 (37.5%)	0.23	*265*
**Regular attendance at daycare**	1 (0.8%)	4 (2.5%)	5 (1.7%)	39 (5.4%)	44 (4.3%)	0.007	*238*
**Central air conditioner in residence**	75 (57.7%)	97 (60.2%)	176 (58.5%)	401 (56.0%)	577 (56.7%)	0.47	*241*
**Clothes dryer in residence**	114 (87.7%)	140 (87.0%)	264 (87.7%)	615 (85.8%)	879 (86.4%)	0.41	*240*
**Use gas cooking stove**	86 (66.2%)	110 (68.3%)	202 (67.1%)	469 (65.4%)	671 (65.9%)	0.60	*240*
**Regular use of air filter**	63 (49.2%)	77 (49.4%)	144 (49.0%)	306 (45.4%)	450 (46.5%)	0.30	*290*
**Residence built before 1950**	20 (15.9%)	23 (14.8%)	45 (15.5%)	107 (15.7%)	152 (15.6%)	0.95	*285*
Environment – ISV							
**Indoor pet(s)**	33 (30.3%)	47 (28.1%)	85 (29.7%)	188 (30.0%)	273 (29.9%)	0.92	*346*
**ETS**	21 (19.3%)	29 (17.3%)	55 (19.2%)	129 (20.5%)	184 (20.1%)	0.64	*342*
**Mom smoked**	12 (11.0%)	15 (8.9%)	29 (10.1%)	74 (11.8%)	103 (11.3%)	0.46	*343*
**Endotoxin - Mother's mattress§**	2.6 (1.0-6.8)	3.5 (1.2-7.6)	3.2 (1.2–7.3)	3.4 (1.3–8.0)	3.5 (1.3–7.8)	0.32	*442*
**Endotoxin - Mother's bedroom floor§**	11.4 (4.9-25.2)	14.5 (6.0-29.3)	13.3 (5.5–28.5)	13.0 (4.8–32.9)	13.1 (5.0–30.6)	0.99	*451*
**Endotoxin - Baby's mattress§**	3.2 (1.4-7.8)	3.6 (1.1-8.3)	3.4 (1.2–8.1)	3.5 (1.3–9.0)	3.35 (1.3–8.7)	0.56	*387*
**Endotoxin - Baby's bedroom floor§**	10.8 (4.0-27.9)	13.6 (5.1-38.7)	12.5 (4.3–31.5)	13.0 (4.5–33.4)	12.9 (4.5–32.9)	0.86	*405*
**Endotoxin - living room floor§**	20.7 (8.0-42.4)	22.2 (8.9-49.3)	22.0 (8.5–46.0)	19.0 (7.6–41.5)	19.5 (7.8–42.4)	0.19	*400*
**Detectable Can f 1**	59 (54.6%)	102 (61.4%)	169 (59.5%)	342 (57.2%)	511 (57.9%)	0.52	*376*
**Detectable Fel d 1**	76 (70.4%)	127 (76.0%)	211 (74.0%)	460 (76.9%)	671 (76.0%)	0.35	*375*
**Detectable Der f 1**	31 (28.7%)	77 (46.1%)†	112 (39.3%)	242 (40.0%)	354 (39.8%)	0.84	*368*
**Regular attendance at daycare**	42 (38.5%)	71 (42.3%)	116 (40.4%)	180 (28.6%)	296 (32.3%)	<.001	*342*
**Central air conditioner in residence**	71 (65.1%)	99 (58.9%)	174 (60.6%)	347 (55.5%)	521 (57.1%)	0.15	*346*
**Clothes dryer in residence**	95 (87.2%)	144 (86.2%)	248 (86.7%)	539 (85.8%)	787 (86.1%)	0.72	*344*
**Use gas cooking stove**	75 (68.8%)	119 (70.8%)	201 (70.0%)	423 (67.4%)	624 (68.2%)	0.42	*343*
**Regular use of air filter**	54 (50.9%)	79 (49.1%)	137 (49.6%)	289 (48.3%)	426 (48.7%)	0.72	*384*
**Residence built before 1950**	22 (20.6%)	17 (10.7%)†	41 (14.9%)	89 (14.9%)	130 (14.9%)	0.98	*386*

Abbreviations: BMI, body mass index; ETS, environmental tobacco smoke; MAAP, the Microbes, Allergy, Asthma, and Pets study; WHEALS, the Wayne County Health Environment Asthma and Allergy Longitudinal Study; NSV, neonatal study visit at 1-month of age; ISV, infant study visit at 6-months of age.

*compares included (n=308) vs excluded (n=950)

† p<0.05 for comparison of NSV to ISV samples

‡ mean (standard deviation)

§ median (interquartile range)

Supplementary Table S2: Description of early life variables used in analyses.

Variable	Measurement Time(s)	Source of Measurement	Additional Details	Rate of Missingness
Demographics				
**Maternal race-ethnicity**	PD/D	Questionnaire	Mother asked what she considers herself to be: African American (non-hispanic, non-arabic), White (non-hispanic, non-arabic), Hispanic, Arabic, other/mixed race	0%
**Marital status**	PD/D	Questionnaire	Current marital status categorized as: Married/living as married; Separated/divorced; Widowed; Never married	0%
**Urban residence**	PD/D	Questionnaire	Using self-reported home address, urban was defined as within the confines of the city of Detroit, all else was defined suburban	0%
**Household income**	PD/D	Questionnaire	Classified in ranges: <$20K; $20K-<$40K; $40K-<$80K; $80K-<$100K; $100K or more	13%
**Maternal education**	PD/D	Questionnaire	Highest level of education categorized as: < high school; High school diploma or equivalent; some college; Bachelor’s degree; Master’s degree or more	0%
**Maternal age**	PD/D	Questionnaire	Calculated from birth date as of PD interview date	0%
**Paternal age**	PD/D	Questionnaire	Calculated from birth date as of PD interview date	9%

Variable	Measurement Time(s)	Source of Measurement	Additional Details	Rate of Missingness
Pregnancy and Delivery				
**Mode of delivery (C-section vs. vaginal)**	PD/D	Chart Abstraction		0%
**Mode of delivery (type)**	PD/D	Chart Abstraction	Specific mode of delivery type: planned C-section, unplanned C-section, vaginal delivery	0%
**Firstborn**	PD/D	Questionnaire	Asked about all previous pregnancies and outcomes	0%
**Parity**	PD/D	Questionnaire	Asked about all previous pregnancies and outcomes	0%
**Number of previous pregnancies**	PD/D	Questionnaire	Asked about all previous pregnancies and outcomes	0%
**Baby gender**	PD/D	Questionnaire	Female; Male	0%
**Maternal antibiotic use during pregnancy**	PD/D	Chart Abstraction	Mom had at least one antibiotic prescription during pregnancy	5%
**Maternal antifungal use during pregnancy**	PD/D	Chart Abstraction	Mom had at least one antifungal prescription during pregnancy	5%
**BMI (first recorded in pregnancy)**	PD/D	Chart Abstraction	Calculated as kg/m^2	5%
**BMI (last recorded in pregnancy)**	PD/D	Chart Abstraction	Calculated as kg/m^2	5%
**Birth weight z-score**	PD/D	Chart Abstraction	Gender- and gestational-age adjusted, calculated using the US population as a reference	5%
**Gestational age**	PD/D	Chart Abstraction		1%
**Season of birth**	PD/D	Questionnaire	Calculated from birth date	0%
**Baby race-ethnicity**	24 Month	Questionnaire	Mother asked what she considers her child to be: African American (non-hispanic, non-arabic), White (non-hispanic, non-arabic), Hispanic, Arabic, other/mixed race.	0%
Feeding Practices				

Variable	Measurement Time(s)	Source of Measurement	Additional Details	Rate of Missingness
**Ever breastfed**	NSV	Questionnaire	Mother asked if she breastfed her baby at any time (even one attempt was considered as ever breastfed)	1%
**Exclusive breastfed**	NSV, ISV	Questionnaire	Currently breastfeeding baby + report of never giving baby any formula	0% (NSV); 0% (ISV)
**Current breastfeeding**	NSV, ISV	Questionnaire	Mother asked if she was breastfeeding her baby at the time of the interview	0% (NSV); 0% (ISV)
**Duration of breastfeeding**	NSV, ISV	Questionnaire	Mother asked when breastfeeding was discontinued	0% (NSV); 7% (ISV)
**Baby had water**	NSV, ISV	Questionnaire	Baby drinks water, excluding rinsing baby's mouth and mixing with formula	0% (NSV); 0% (ISV)
**Baby had antibiotic(s)**	NSV, ISV	Chart Abstraction	Any non-topical antibiotics prescribed, before the respective stool sample was collected	12% (NSV); 22% (ISV)
**Early solid food (<4 months of age)**	ISV	Questionnaire	Any cereal, solid food, or cow's milk (not processed in a formula) introduced before 4 months of age	0%
Environment				
**Indoor pet(s)**	PD/D, NSV, ISV	Questionnaire	Any dog(s) or cat(s) in household that spent at least one hour inside the home every day	0% (PD/D); 0% (NSV); 1% (ISV)
**ETS**	PD/D, NSV, ISV	Questionnaire	Any person living in the home who smoked at least 1 cigarette/cigar/pipe per day	0% (PD/D); 0% (NSV); 0% (ISV)
**Mom smoked**	PD/D, NSV, ISV	Questionnaire	Mom smoked at least 1 cigarette/cigar/pipe per day	0% (PD/D); 0% (NSV); 0% (ISV)
**Endotoxin - Mother's mattress**	NSV, ISV	Home Visit	Measured from collected house dust samples*	5% (NSV); 5% (ISV)

Variable	Measurement Time(s)	Source of Measurement	Additional Details	Rate of Missingness
**Endotoxin - Mother's bedroom floor**	NSV, ISV	Home Visit	Measured from collected house dust samples*	4% (NSV); 6% (ISV)
**Endotoxin - Baby's mattress**	NSV, ISV	Home Visit	Measured from collected house dust samples*	2% (NSV); 2% (ISV)
**Endotoxin - Baby's bedroom floor**	NSV, ISV	Home Visit	Measured from collected house dust samples*	3% (NSV); 4% (ISV)
**Endotoxin - living room floor**	NSV, ISV	Home Visit	Measured from collected house dust samples*	3% (NSV); 4% (ISV)
**Detectable Can f 1**	NSV, ISV	Home Visit	Measured from collected house dust samples, only from child's bedroom floor*	3% (NSV); 1% (ISV)
**Detectable Fel d 1**	NSV, ISV	Home Visit	Measured from collected house dust samples, only from child's bedroom floor*	2% (NSV); 1% (ISV)
**Detectable Der f 1**	NSV, ISV	Home Visit	Measured from collected house dust samples, only from child's bedroom floor*	2% (NSV); 1% (ISV)
**Regular attendance at daycare**	NSV, ISV	Questionnaire	Mom reports if child spends at least 3 days per week or at least 10 days per month in daycare	0% (NSV); 0% (ISV)
**Central air conditioner in residence**	NSV, ISV	Questionnaire	Maternal report of central air conditioning in home	0% (NSV); 0% (ISV)
**Clothes dryer in residence**	NSV, ISV	Questionnaire	Maternal report of clothes' dryer in home	0% (NSV); 1% (ISV)
**Use gas cooking stove**	NSV, ISV	Questionnaire	Maternal report of use of gas cooking store, range or oven	0% (NSV); 0% (ISV)
**Regular use of air filter**	NSV, ISV	Questionnaire	Regular use defined as at least once a month	2% (NSV); 4% (ISV)
**Year residence built**	NSV, ISV	Questionnaire	Maternal report categorized as: 1990-Present; 1980-1989; 1970-1979; 1950-1969; Before 1950	0% (NSV); 3% (ISV)
Specimen Characteristics				

Variable	Measurement Time(s)	Source of Measurement	Additional Details	Rate of Missingness
**Age at stool collection (days)**	NSV, ISV	Home Visit	Calculated by subtracting baby's date of birth from stool collection date	0% (NSV); 0% (ISV)
**Season stool collected**	NSV, ISV	Home Visit	Calculated from stool collection date	0% (NSV); 0% (ISV)

Abbreviations: BMI, body mass index; ETS, environmental tobacco smoke; PD/D, pre-delivery/delivery; NSV, neonatal study visit at 1-month of age; ISV, infant study visit at 6-months of age.
*See methods for additional details

Supplementary Table S3: Single factor PERMANOVA gut microbiome compositional analyses using both unweighted and weighted UniFrac distances metrics.

Factor	Neonates		Infants	
	Unweighted UniFrac	Weighted UniFrac	Unweighted UniFrac	Weighted UniFrac
	P (% variation explained)			
**Demographics**				
Maternal race-ethnicity	0.002 (3.8%)*	0.478 (3%)	<0.001 (3.3%)	0.028 (3.9%)
Marital status	0.001 (2.2%)	0.247 (1.9%)	0.001 (2.3%)	0.443 (1.8%)
Urban residence	0.147 (0.9%)	0.221 (1%)	0.008 (0.8%)	0.18 (0.9%)
Household income	0.008 (4.1%)	0.077 (5.2%)	0.001 (3.5%)	0.020 (4.8%)
Maternal education	0.044 (2.6%)	0.385 (2.4%)	<0.001 (2.8%)	0.072 (2.7%)
Maternal age	0.15 (0.9%)	0.36 (0.8%)	0.492 (0.6%)	0.395 (0.6%)
Paternal age	0.22 (0.9%)	0.208 (1.2%)	0.749 (0.6%)	0.497 (0.6%)
**Pregnancy and Delivery**				
Mode of delivery (c-section vs. vaginal)	<0.001 (1.9%)	0.002 (3.4%)	0.001 (1%)	0.11 (1%)
Mode of delivery (type)ł	<0.001 (2.7%)	0.013 (3.6%)	0.001 (1.7%)	0.018 (2.5%)
Vaginal vs. planned c-section	<0.001 (1.8%)	0.017 (2.7%)	0.072 (1.0%)	0.31 (0.9%)
Vaginal vs. unplanned c-section	0.001 (1.6%)	0.031 (2.2%)	0.001 (1.3%)	0.019 (2.0%)
Planned vs. unplanned c-section	0.48 (2.5%)	0.96 (0.7%)	0.15 (1.5%)	0.022 (3.5%)
Firstborn	0.002 (1.2%)	0.039 (1.8%)	0.015 (0.8%)	0.147 (0.9%)
Parity	0.074 (0.9%)	0.199 (1.1%)	0.019 (0.8%)	0.24 (0.8%)
Number of previous pregnancies	0.004 (1.1%)	0.135 (1.3%)	0.022 (0.8%)	0.251 (0.7%)
Baby gender	0.298 (0.8%)	0.696 (0.5%)	0.219 (0.6%)	0.823 (0.3%)
Maternal antibiotic use during pregnancy	0.146 (0.9%)	0.26 (1%)	0.519 (0.6%)	0.208 (0.9%)
Maternal antifungal use during pregnancy	0.658 (0.8%)	0.757 (0.4%)	0.066 (0.8%)	0.686 (0.4%)
BMI (first recorded in pregnancy)	0.004 (1.2%)	0.602 (0.6%)	0.001 (1.1%)	0.192 (0.9%)
BMI (last recorded in pregnancy)	0.026 (1%)	0.505 (0.7%)	0.001 (1.1%)	0.246 (0.8%)
Birth weight z-score	0.951 (0.7%)	0.317 (0.9%)	0.495 (0.6%)	0.739 (0.4%)
Gestational age	0.523 (0.8%)	0.495 (0.7%)	0.383 (0.6%)	0.011 (1.9%)
Season of birth	0.409 (2.3%)	0.087 (3.5%)	0.78 (1.7%)	0.079 (2.7%)
Baby race-ethnicity	0.006 (3.7%)	0.902 (2%)	<0.001 (3.3%)	0.04 (3.8%)
Mom smoked during pregnancy	0.396 (0.8%)	0.905 (0.3%)	0.185 (0.7%)	0.047 (1.3%)
**Feeding Practices – NSV**				
Ever breastfed	<0.001 (1.4%)	0.284 (0.9%)	0.008 (0.9%)	0.191 (0.8%)
Exclusive breastfed	<0.001 (1.4%)	0.049 (1.7%)	<0.001 (1.7%)	<0.001 (5.1%)
Current breastfeeding	<0.001 (2.1%)	0.224 (1%)	<0.001 (1.3%)	0.004 (2.2%)
Duration of breastfeeding	0.009 (1.1%)	0.613 (0.6%)	0.017 (0.9%)	0.212 (0.9%)
Baby had water	0.004 (1.2%)	0.206 (1.1%)	0.185 (0.7%)	0.657 (0.4%)

Factor	Neonates Unweighted UniFrac	Neonates Weighted UniFrac	Infants Unweighted UniFrac	Infants Weighted UniFrac
	P (% variation explained)			
Baby had antibiotic(s)	0.134 (1%)	0.623 (0.6%)	0.292 (1.2%)	0.375 (1.2%)
**Feeding Practices – ISV**				
Exclusive breastfed	—‡	—	<0.001 (2%)	<0.001 (7.1%)
Current breastfeeding	—	—	<0.001 (2.6%)	<0.001 (9.2%)
Duration of breastfeeding	—	—	<0.001 (1.8%)	<0.001 (3.8%)
Baby had water	—	—	0.035 (0.8%)	0.311 (0.7%)
Baby had antibiotic(s)	—	—	0.395 (0.8%)	0.743 (0.5%)
Early solid food (<4 months of age)	—	—	0.733 (0.6%)	0.148 (0.9%)
**Environment – during pregnancy**				
Indoor pet(s) during pregnancy	0.065 (2.6%)	0.312 (2.6%)	0.132 (1.9%)	0.17 (2.3%)
ETS during pregnancy	0.021 (1%)	0.314 (0.9%)	0.223 (0.6%)	0.169 (0.9%)
**Environment – NSV**				
Indoor pet(s)	0.086 (2.6%)	0.391 (2.4%)	0.063 (2.1%)	0.111 (2.6%)
ETS	0.007 (1.1%)	0.328 (0.9%)	0.388 (0.6%)	0.274 (0.8%)
Mom smoked	0.321 (0.8%)	0.475 (0.7%)	0.147 (0.7%)	0.171 (0.9%)
Endotoxin - Mother's mattress	0.156 (0.9%)	0.081 (1.6%)	0.262 (0.7%)	0.729 (0.4%)
Endotoxin - Mother's bedroom floor	0.646 (0.8%)	0.944 (0.2%)	0.242 (0.7%)	0.634 (0.5%)
Endotoxin - Baby's mattress	0.315 (0.8%)	0.108 (1.4%)	0.894 (0.6%)	0.996 (0.1%)
Endotoxin - Baby's bedroom floor	0.25 (0.9%)	0.926 (0.3%)	0.242 (0.7%)	0.326 (0.7%)
Endotoxin - living room floor	0.133 (0.9%)	0.094 (1.4%)	0.282 (0.7%)	0.357 (0.7%)
Detectable Can f 1	0.175 (0.9%)	0.28 (1%)	0.313 (0.7%)	0.265 (0.8%)
Detectable Fel d 1	0.154 (0.9%)	0.813 (0.4%)	0.088 (0.8%)	0.479 (0.6%)
Detectable Der f 1	0.386 (0.8%)	0.711 (0.5%)	0.815 (0.6%)	0.823 (0.3%)
Regular attendance at daycare	0.856 (0.7%)	0.932 (0.3%)	0.324 (0.6%)	0.858 (0.3%)
Central air conditioner in residence	0.185 (0.8%)	0.369 (0.8%)	0.017 (0.8%)	0.145 (1%)
Clothes dryer in residence	0.141 (0.9%)	0.791 (0.4%)	0.539 (0.6%)	0.701 (0.4%)
Use gas cooking stove	0.279 (0.8%)	0.18 (1.1%)	0.641 (0.6%)	0.63 (0.4%)
Regular use of air filter	0.057 (1%)	0.136 (1.3%)	0.001 (1.1%)	0.019 (1.7%)
Year residence built	0.345 (1.6%)	0.461 (1.5%)	0.953 (1.1%)	0.911 (0.7%)
**Environment – ISV**				
Indoor pet(s)	—	—	0.339 (0.6%)	0.299 (0.7%)
ETS	—	—	0.167 (0.7%)	0.115 (1%)
Endotoxin - Mother's mattress	—	—	0.54 (0.6%)	0.737 (0.4%)

Factor	Neonates		Infants	
	Unweighted UniFrac	Weighted UniFrac	Unweighted UniFrac	Weighted UniFrac
	P (% variation explained)			
Endotoxin - Mother's bedroom floor	—	—	0.368 (0.7%)	0.435 (0.6%)
Endotoxin - Baby's mattress	—	—	0.923 (0.5%)	0.95 (0.2%)
Endotoxin - Baby's bedroom floor	—	—	0.791 (0.6%)	0.714 (0.4%)
Endotoxin - living room floor	—	—	0.83 (0.6%)	0.879 (0.3%)
Mom smoked	—	—	0.122 (0.7%)	0.379 (0.6%)
Detectable Can f 1	—	—	0.274 (0.6%)	0.228 (0.8%)
Detectable Fel d 1	—	—	0.33 (0.6%)	0.031 (1.4%)
Detectable Der f 1	—	—	0.525 (0.6%)	0.212 (0.8%)
Regular attendance at daycare	—	—	0.08 (0.7%)	0.167 (0.9%)
Central air conditioner in residence	—	—	0.002 (0.9%)	0.019 (1.6%)
Clothes dryer in residence	—	—	0.355 (0.6%)	0.218 (0.8%)
Use gas cooking stove	—	—	0.396 (0.6%)	0.676 (0.4%)
Regular use of air filter	—	—	0.058 (0.8%)	0.336 (0.7%)
Year residence built	—	—	0.996 (1.1%)	0.993 (0.4%)
**Specimen Characteristics**				
Age at stool collection (days)	<0.001 (1.5%)	0.097 (1.4%)	<0.001 (1.1%)	0.022 (1.5%)
Season stool collected	0.195 (2.5%)	0.182 (3%)	0.492 (1.8%)	0.368 (1.9%)

Abbreviations: BMI, body mass index; ETS, environmental tobacco smoke; NSV, neonatal study visit at 1-month of age; ISV, infant study visit at 6-months of age.

*factor/metric combinations with PERMANOVA p-value < 0.05 are shaded.

ƚ planned C-section, unplanned C-section, vaginal delivery

‡ indicates that the analysis was not conducted among the neonates because the measurement corresponded to the one taken at the ISV.

Supplementary Table S4: Analysis of single factors and gut microbiome gross community measures (richness, evenness, diversity) for both MAAP neonates and infants.

		Neonates						Infants					
		**Richness**		**Evenness**		**Diversity**		**Richness**		**Evenness**		**Diversity**	
Factor		N, Mean (SD)	P*	N, Mean (SD)	P	N, Mean (SD)	P	N, Mean (SD)	P	N, Mean (SD)	P	N, Mean (SD)	P
**Demographics**													
Maternal race-ethnicity	Caucasian	31, 972.94 (389.87)	0.061	31, 0.35 (0.08)	0.047	31, 50.86 (16.2)	0.027	48, 1418.44 (664.11)	<0.001	48, 0.39 (0.1)	0.001	48, 69.65 (23.46)	<0.001
	Afr Amer	82, 1027.59 (424.67)		82, 0.37 (0.07)		82, 53.72 (17.36)		100, 1892.09 (684.26)		100, 0.45 (0.08)		100, 88.26 (24.18)	
	Hispanic	8, 925.5 (617.25)		8, 0.34 (0.14)		8, 49.49 (24.86)		6, 1854.33 (946.77)		6, 0.45 (0.09)		6, 84.91 (35.16)	
	Arab Am	5, 1020.6 (695.37)		5, 0.34 (0.13)		5, 54.65 (22.09)		9, 1243.33 (516.84)		9, 0.38 (0.07)		9, 63.45 (18.82)	
	Other	4, 541 (175.28)		4, 0.26 (0.04)		4, 30.34 (5.67)		5, 1594 (693.91)		5, 0.39 (0.08)		5, 72.45 (20.49)	
Marital status	Married	75, 891.05 (390.39)	0.001	75, 0.34 (0.08)	0.001	75, 47.66 (16.06)	0.001	114, 1559.57 (643.1)	0.001	114, 0.41 (0.09)	0.013	114, 75.74 (23.4)	0.001
	Divorced/Separated	6, 835.83 (143.12)		6, 0.35 (0.06)		6, 47.36 (7.2)		6, 1774 (891.82)		6, 0.46 (0.07)		6, 84.15 (32.73)	
	Widowed	0, NA (NA)		0, NA (NA)		0, NA (NA)		1, 1497 (NA)		1, 0.44 (NA)		1, 75.74 (NA)	
	Never Married	49, 1168.37 (479.01)		49, 0.39 (0.07)		49, 59.46 (18.98)		47, 2077.62 (744)		47, 0.46 (0.08)		47, 93.56 (26.07)	
Urban residence	No	59, 911.95 (376.01)	0.073	59, 0.34 (0.08)	0.006	59, 48.93 (15.47)	0.096	85, 1574.79 (634.64)	0.021	85, 0.41 (0.09)	0.015	85, 75.82 (23.42)	0.013
	Yes	71, 1060.41 (476.88)		71, 0.37 (0.07)		71, 54.72 (19.26)		83, 1852.08 (765.99)		83, 0.44 (0.09)		83, 86.35 (26.69)	
Household income	<20K	16, 1013.81 (327.77)	0.12	16, 0.37 (0.06)	0.061	16, 53.91 (12.48)	0.28	18, 1693.72 (552.98)	0.49	18, 0.44 (0.06)	0.19	18, 80.27 (18.53)	0.34
	20-<40K	29, 1136.17 (472.32)		29, 0.38 (0.06)		29, 57.59 (19.41)		32, 1856.28 (806.1)		32, 0.45 (0.08)		32, 87.46 (29.51)	
	40-<80K	31, 912.81 (508.04)		31, 0.33 (0.09)		31, 49.36 (20.24)		40, 1697.4 (693.82)		40, 0.43 (0.09)		40, 80.58 (25.51)	
	80-<100K	16, 1064.19 (402.43)		16, 0.37 (0.08)		16, 53.91 (17.74)		30, 1578 (717.44)		30, 0.41 (0.1)		30, 75.51 (25.04)	
	>=100K	21, 950.74 (369.11)		21, 0.36 (0.08)		21, 50.17 (15.98)		22, 1559.04 (630.51)		22, 0.39 (0.09)		22, 75.04 (22.49)	
Maternal education	Some HS	5, 901 (475.6)	0.067	5, 0.37 (0.07)	0.173	5, 48.15 (21.92)	0.013	7, 1449.57 (915.14)	0.003	7, 0.41 (0.1)	0.028	7, 71.41 (37.52)	0.001
	HS or equiv	23, 1118.65 (439.96)		23, 0.37 (0.08)		23, 58.16 (15.74)		21, 1941.86 (732.04)		21, 0.45 (0.08)		21, 89.18 (26.37)	
	Some College	60, 1041.45 (474.97)		60, 0.36 (0.09)		60, 54.15 (19.23)		79, 1848.32 (685.47)		79, 0.44 (0.08)		79, 86.77 (23.49)	
	Bachelors or more	42, 866.02 (353.79)		42, 0.34 (0.08)		42, 46.3 (14.93)		61, 1485.85 (665.87)		61, 0.4 (0.1)		61, 71.88 (23.8)	
Maternal age ł	---	130, -0.17	0.047	130, -0.13	0.126	130, -0.16	0.061	168, -0.1	0.218	168, -0.06	0.448	168, -0.08	0.304
Paternal age ł	---	118, -0.15	0.11	118, -0.13	0.17	118, -0.15	0.101	153, -0.02	0.784	153, 0	0.967	153, -0.01	0.919

		Neonates						Infants					
		Richness		Evenness		Diversity		Richness		Evenness		Diversity	
Factor		N, Mean (SD)	P*	N, Mean (SD)	P	N, Mean (SD)	P	N, Mean (SD)	P	N, Mean (SD)	P	N, Mean (SD)	P
**Pregnancy and Delivery**													
Mode of delivery (c-section vs. vaginal)	Vaginal	89, 1016.43 (482.13)	0.739	89, 0.36 (0.08)	0.11	89, 53.85 (19.58)	0.216	94, 1653.02 (743.17)	0.192	94, 0.42 (0.09)	0.332	94, 79.22 (26.9)	0.306
	C-Section	41, 942.24 (324.77)		41, 0.34 (0.07)		41, 48.27 (12.57)		74, 1786.43 (673.11)		74, 0.43 (0.08)		74, 83.31 (23.74)	
Mode of delivery (type)	Vaginal	89, 1016.43 (482.13)	0.927	89, 0.36 (0.08)	0.263	89, 53.85 (19.58)	0.45	94, 1653.02 (743.17)	0.247	94, 0.42 (0.09)	0.231	94, 79.22 (26.9)	0.289
	Planned Csect	17, 939.65 (288.5)		17, 0.34 (0.08)		17, 48.07 (11.19)		32, 1903.47 (717.41)		32, 0.45 (0.07)		32, 87.96 (25.23)	
	Unplanned Csect	24, 944.08 (354.3)		24, 0.34 (0.07)		24, 48.42 (13.7)		42, 1697.26 (631.44)		42, 0.42 (0.08)		42, 79.77 (22.18)	
Firstborn	No	85, 1042.29 (448.76)	0.062	85, 0.36 (0.08)	0.434	85, 54.53 (17.7)	0.013	92, 1700.67 (685.53)	0.911	92, 0.42 (0.09)	0.978	92, 81.2 (24.58)	0.622
	Yes	45, 899.98 (407.78)		45, 0.35 (0.08)		45, 47.49 (17.28)		76, 1725.24 (751.74)		76, 0.43 (0.08)		76, 80.82 (26.87)	
Parity ł	---	130, 0.12	0.158	130, 0.04	0.652	130, 0.19	0.032	168, -0.04	0.603	168, -0.05	0.496	168, 0	0.959
Number of previous pregnancies	0	29, 794.62 (236.18)	0.045	29, 0.33 (0.08)	0.316	29, 43.46 (11.63)	0.016	47, 1628.68 (720.7)	0.197	47, 0.42 (0.09)	0.197	47, 77.14 (24.43)	0.217
	1	30, 1059.13 (407.2)		30, 0.36 (0.09)		30, 54.83 (17)		40, 1785.28 (758.7)		40, 0.43 (0.09)		40, 83.04 (28.84)	
	2	31, 970.87 (499.16)		31, 0.36 (0.08)		31, 51.64 (19.71)		40, 1784.78 (753.5)		40, 0.43 (0.09)		40, 83.68 (26.34)	
	3	22, 1090.82 (497.94)		22, 0.36 (0.09)		22, 55.59 (19.82)		22, 1855.09 (613.16)		22, 0.45 (0.07)		22, 87.75 (20.42)	
	4 or more	18, 1121.17 (484.04)		18, 0.38 (0.06)		18, 57.93 (18.09)		19, 1443.05 (587.7)		19, 0.39 (0.1)		19, 73.03 (23.64)	
Baby gender	Male	71, 975.61 (385.46)	0.89	71, 0.35 (0.08)	0.607	71, 51.76 (16.5)	0.826	86, 1791 (734.12)	0.109	86, 0.43 (0.09)	0.543	86, 83.46 (26.39)	0.141
	Female	59, 1014 (497.94)		59, 0.36 (0.08)		59, 52.49 (19.41)		82, 1628.71 (687.27)		82, 0.42 (0.08)		82, 78.48 (24.57)	
Maternal antibiotic use during pregnancy	No	57, 1004.44 (523.15)	0.559	57, 0.35 (0.09)	0.772	57, 52.4 (21.48)	0.379	72, 1629.43 (734.39)	0.167	72, 0.41 (0.09)	0.089	72, 77.79 (26.48)	0.191
	Yes	68, 982.97 (366.78)		68, 0.36 (0.07)		68, 51.8 (14.41)		85, 1761.36 (688.75)		85, 0.43 (0.09)		85, 82.87 (24.14)	
Maternal antifungal use during pregnancy	No	104, 1005.07 (446.66)	0.44	104, 0.36 (0.08)	0.59	104, 52.79 (18.22)	0.343	121, 1633.14 (685.63)	0.057	121, 0.41 (0.09)	0.039	121, 77.87 (24.69)	0.033
	Yes	21, 931.81 (430.74)		21, 0.35 (0.08)		21, 48.5 (16.21)		36, 1928.47 (755.68)		36, 0.45 (0.07)		36, 89.52 (25.52)	

		Neonates						Infants					
		Richness		Evenness		Diversity		Richness		Evenness		Diversity	
Factor		N, Mean (SD)	P*	N, Mean (SD)	P	N, Mean (SD)	P	N, Mean (SD)	P	N, Mean (SD)	P	N, Mean (SD)	P
BMI (first recorded in pregnancy) ᵻ	---	124, 0.21	0.018	124, 0.08	0.403	124, 0.24	0.006	158, 0.23	0.003	158, 0.22	0.007	158, 0.25	0.002
BMI (last recorded in pregnancy) ᵻ	---	124, 0.18	0.042	124, 0.06	0.53	124, 0.21	0.018	158, 0.23	0.003	158, 0.19	0.016	158, 0.24	0.002
Birth weight z-score ᵻ	---	124, -0.02	0.815	124, -0.08	0.384	124, -0.02	0.833	158, 0.01	0.933	158, -0.02	0.85	158, -0.01	0.949
Gestational age ᵻ	---	130, -0.19	0.027	130, -0.23	0.009	130, -0.19	0.031	165, -0.06	0.465	165, -0.13	0.087	165, -0.06	0.434
Season of birth	Winter	36, 972.86 (400.81)	0.355	36, 0.36 (0.08)	0.861	36, 51.47 (17.78)	0.492	21, 1739.19 (786.35)	0.873	21, 0.43 (0.09)	0.946	21, 81.77 (30.27)	0.925
	Spring	29, 928.86 (430.15)		29, 0.35 (0.09)		29, 49.73 (16.1)		39, 1700.67 (640.98)		39, 0.42 (0.07)		39, 80.83 (22.9)	
	Summer	31, 952.84 (396.39)		31, 0.35 (0.08)		31, 50.53 (16.2)		52, 1742.6 (694.15)		52, 0.43 (0.1)		52, 81.6 (25.75)	
	Fall	34, 1105.76 (513.32)		34, 0.37 (0.08)		34, 56.19 (20.52)		56, 1680.64 (768.68)		56, 0.42 (0.09)		56, 80.35 (25.94)	
Baby race-ethnicity	Caucasian	25, 922.84 (343.8)	0.144	25, 0.34 (0.08)	0.185	25, 49.4 (15.47)	0.07	40, 1364.2 (637.81)	<0.001	40, 0.38 (0.1)	<0.001	40, 67.84 (23.13)	<0.001
	Afr Amer	83, 1029.52 (424.13)		83, 0.37 (0.07)		83, 53.77 (17.32)		102, 1898.33 (688.36)		102, 0.45 (0.08)		102, 88.36 (24.29)	
	Hispanic	11, 1053.09 (634.91)		11, 0.34 (0.13)		11, 53.5 (24.71)		9, 1649.44 (831.58)		9, 0.44 (0.08)		9, 78.01 (29.89)	
	Arab Am	5, 1020.6 (695.37)		5, 0.34 (0.13)		5, 54.65 (22.09)		12, 1286 (470.09)		12, 0.38 (0.06)		12, 64.51 (17.22)	
	Other	6, 647.67 (238.25)		6, 0.32 (0.07)		6, 35.43 (8.89)		5, 1821 (842.14)		5, 0.43 (0.1)		5, 81.91 (24.97)	
Mom smoked during pregnancy	No	117, 984.32 (445.8)	0.235	117, 0.35 (0.08)	0.156	117, 51.87 (18.19)	0.406	152, 1704.15 (709.06)	0.742	152, 0.42 (0.09)	0.128	152, 80.88 (25.28)	0.827
	Yes	13, 1071.46 (374.92)		13, 0.39 (0.06)		13, 54.05 (14.4)		16, 1784.31 (781.58)		16, 0.45 (0.08)		16, 82.38 (28.98)	
**Feeding Practices – NSV**													
Ever breastfed	No	29, 1202.41 (415.34)	<0.001	29, 0.39 (0.06)	0.009	29, 61.06 (17.75)	0.001	37, 1962.95 (740.59)	0.011	37, 0.44 (0.1)	0.177	37, 91.07 (26.65)	0.003
	Yes	101, 932.91 (428.51)		101, 0.35 (0.08)		101, 49.52 (17.06)		129, 1630.65 (672.15)		129, 0.42 (0.08)		129, 77.86 (24.12)	
Exclusive breastfed	No	114, 1032.75 (448.13)	0.002	114, 0.37 (0.07)	0.005	114, 53.82 (18.11)	0.001	131, 1825.56 (692.84)	<0.001	131, 0.44 (0.08)	<0.001	131, 85.13 (24.76)	<0.001
	Yes	16, 710.06 (212.12)		16, 0.29 (0.11)		16, 39.76 (8.48)		30, 1195.17 (535.27)		30, 0.36 (0.09)		30, 62.5 (20.68)	

		Neonates						Infants					
		**Richness**		**Evenness**		**Diversity**		**Richness**		**Evenness**		**Diversity**	
Factor		N, Mean (SD)	P*	N, Mean (SD)	P	N, Mean (SD)	P	N, Mean (SD)	P	N, Mean (SD)	P	N, Mean (SD)	P
Current breastfeeding	No	60, 1196.02 (488.81)	<0.001	60, 0.39 (0.07)	<0.001	60, 60.45 (19.37)	<0.001	70, 1929.91 (700.42)	<0.001	70, 0.45 (0.08)	0.002	70, 89.02 (25.15)	<0.001
	Yes	70, 819.04 (298.32)		70, 0.32 (0.08)		70, 44.92 (12.62)		91, 1537.46 (670.56)		91, 0.4 (0.09)		91, 74.68 (24.23)	
Duration of breastfeeding ɫ	---	130, -0.27	0.002	130, -0.24	0.007	130, -0.26	0.003	160, -0.24	0.003	160, - 0.17	0.028	160, - 0.25	0.001
Baby had water	No	97, 950.09 (455.33)	0.005	97, 0.35 (0.08)	0.034	97, 50.33 (18.61)	0.007	132, 1667.84 (707.26)	0.119	132, 0.42 (0.09)	0.173	132, 79.32 (25.62)	0.106
	Yes	33, 1119.24 (363.21)		33, 0.38 (0.06)		33, 57.27 (14.22)		29, 1891.31 (699.43)		29, 0.45 (0.07)		29, 88.18 (24.44)	
Baby had antibiotic(s)	No	112, 981.53 (432.1)	0.048	112, 0.35 (0.08)	0.022	112, 51.74 (17.78)	0.112	85, 1791.13 (691.93)	0.398	85, 0.43 (0.09)	0.324	85, 83.99 (24.62)	0.408
	Yes	3, 1444.33 (312.74)		3, 0.46 (0.05)		3, 66.45 (11)		5, 1532 (935.46)		5, 0.39 (0.1)		5, 75.09 (37.12)	
**Feeding Practices – ISV**													
Exclusive breastfed	No							152, 1797.18 (691.59)	<0.001	152, 0.44 (0.08)	<0.00 1	152, 84.08 (24.61)	<0.001
	Yes							16, 900.56 (295.92)		16, 0.32 (0.07)		16, 51.97 (13.53)	
Current breastfeeding	No							122, 1905.52 (698.9)	<0.001	122, 0.45 (0.08)	<0.00 1	122, 88 (24.35)	<0.001
	Yes							46, 1197.96 (455.75)		46, 0.36 (0.08)		46, 62.52 (18.67)	
Duration of breastfeeding ɫ	---							157, -0.4	<0.001	157, - 0.34	<0.00 1	157, - 0.41	<0.001
Baby had water	No							53, 1550.38 (653.86)	0.054	53, 0.41 (0.09)	0.044	53, 74.63 (23.87)	0.036
	Yes							115, 1786.17 (731.05)		115, 0.43 (0.09)		115, 83.97 (25.88)	
Baby had antibiotic(s)	No							102, 1799.41 (734.64)	0.304	102, 0.43 (0.09)	0.684	102, 84.41 (25.6)	0.188
	Yes							29, 1621.59 (702.87)		29, 0.42 (0.09)		29, 76.89 (25.42)	
Early solid food (<4 months of age)	No							97, 1680.91 (711.76)	0.462	97, 0.42 (0.09)	0.357	97, 79.94 (25.45)	0.48
	Yes							71, 1753.97 (720.34)		71, 0.43 (0.09)		71, 82.5 (25.82)	
**Environment – during pregnancy**													

		Neonates						Infants					
		**Richness**		**Evenness**		**Diversity**		**Richness**		**Evenness**		**Diversity**	
Factor		N, Mean (SD)	P*	N, Mean (SD)	P	N, Mean (SD)	P	N, Mean (SD)	P	N, Mean (SD)	P	N, Mean (SD)	P
Indoor pet(s) during pregnancy	Neither	81, 1014.14 (479.35)	0.613	81, 0.36 (0.08)	0.042	81, 53.01 (19.08)	0.48	106, 1775.41 (748.91)	0.343	106, 0.43 (0.09)	0.21	106, 83.1 (26.59)	0.317
	Dog only	31, 930.65 (410.4)		31, 0.33 (0.08)		31, 48.94 (17.28)		33, 1729.82 (684.7)		33, 0.44 (0.08)		33, 82.71 (24.59)	
	Cat only	13, 1033.77 (294.11)		13, 0.38 (0.06)		13, 54.86 (12.82)		14, 1471.43 (466.83)		14, 0.4 (0.06)		14, 73.47 (18.4)	
	Both	5, 932 (199.48)		5, 0.39 (0.05)		5, 49.49 (9.29)		15, 1446.87 (662.68)		15, 0.4 (0.09)		15, 69.71 (23.71)	
ETS during pregnancy	No	97, 951.89 (448.33)	0.011	97, 0.35 (0.09)	0.029	97, 50.32 (18.29)	0.007	125, 1656.92 (672.68)	0.135	125, 0.42 (0.08)	0.031	125, 79.5 (24.35)	0.213
	Yes	33, 1113.97 (390.76)		33, 0.38 (0.06)		33, 57.29 (15.42)		43, 1871.28 (810.56)		43, 0.45 (0.09)		43, 85.47 (28.65)	
**Environment – NSV**													
Indoor pet(s)	Neither	86, 1012.47 (481.27)	0.639	86, 0.36 (0.09)	0.052	86, 53.15 (19.47)	0.553	111, 1767.97 (725.76)	0.181	111, 0.43 (0.09)	0.114	111, 82.83 (26.25)	0.149
	Dog only	28, 923.79 (378.58)		28, 0.33 (0.07)		28, 48 (14.65)		27, 1698.11 (691.42)		27, 0.43 (0.08)		27, 81.86 (24.21)	
	Cat only	10, 1005.9 (284.53)		10, 0.38 (0.06)		10, 53.49 (12.55)		12, 1534.5 (454.49)		12, 0.41 (0.06)		12, 76.08 (17.48)	
	Both	6, 1016.17 (272.65)		6, 0.39 (0.05)		6, 53.71 (13.25)		11, 1317.73 (730.23)		11, 0.37 (0.1)		11, 64.5 (25.52)	
ETS	No	106, 970.41 (454.02)	0.045	106, 0.35 (0.08)	0.215	106, 50.9 (18.2)	0.02	135, 1706.9 (714.89)	0.701	135, 0.42 (0.09)	0.863	135, 80.83 (25.57)	0.593
	Yes	24, 1092.96 (354.54)		24, 0.37 (0.06)		24, 57.36 (15.19)		26, 1714.27 (690.76)		26, 0.42 (0.1)		26, 81.34 (26.06)	
Mom smoked	No	116, 993.44 (457.25)	0.424	116, 0.35 (0.08)	0.208	116, 52.12 (18.49)	0.478	149, 1702.93 (724.67)	0.535	149, 0.42 (0.09)	0.27	149, 80.75 (26.11)	0.591
	Yes	14, 989.64 (245.68)		14, 0.38 (0.04)		14, 51.82 (11.05)		12, 1772.17 (489.67)		12, 0.45 (0.06)		12, 82.92 (18.15)	
Endotoxin - Mother's mattress ł	---	123, -0.05	0.615	123, -0.04	0.684	123, -0.04	0.688	152, -0.08	0.336	152, -0.02	0.825	152, -0.08	0.347
Endotoxin - Mother's bedroom floor ł	---	125, 0.18	0.05	125, 0.18	0.049	125, 0.19	0.037	149, -0.11	0.177	149, -0.04	0.664	149, -0.1	0.244
Endotoxin - Baby's mattressł	---	127, 0.02	0.836	127, -0.04	0.626	127, 0.04	0.632	154, 0.05	0.559	154, 0.11	0.177	154, 0.06	0.477
Endotoxin - Baby's bedroom floor ł	---	126, 0.21	0.021	126, 0.18	0.047	126, 0.23	0.01	152, -0.04	0.649	152, 0	0.993	152, -0.02	0.79

		Neonates						Infants					
		**Richness**		**Evenness**		**Diversity**		**Richness**		**Evenness**		**Diversity**	
Factor		N, Mean (SD)	P*	N, Mean (SD)	P	N, Mean (SD)	P	N, Mean (SD)	P	N, Mean (SD)	P	N, Mean (SD)	P
Endotoxin - living room floor ɫ	---	126, 0.04	0.672	126, 0.03	0.736	126, 0.05	0.56	155, -0.02	0.847	155, -0.01	0.865	155, -0.01	0.937
Detectable Can f 1	No	47, 923.02 (401.8)	0.142	47, 0.35 (0.08)	0.709	47, 48.9 (16.22)	0.141	62, 1811.13 (695.77)	0.102	62, 0.44 (0.08)	0.151	62, 84.36 (24.91)	0.188
	Yes	79, 1038.3 (466.18)		79, 0.36 (0.08)		79, 53.97 (18.93)		94, 1639.12 (708.98)		94, 0.41 (0.09)		94, 78.61 (25.49)	
Detectable Fel d 1	No	44, 911.16 (326.11)	0.245	44, 0.34 (0.06)	0.132	44, 48.8 (14.37)	0.282	37, 1971.19 (788.63)	0.029	37, 0.45 (0.08)	0.079	37, 91.04 (28.59)	0.023
	Yes	83, 1040.19 (490.37)		83, 0.36 (0.09)		83, 53.86 (19.51)		117, 1626.4 (666.81)		117, 0.41 (0.09)		117, 77.8 (23.68)	
Detectable Der f 1	No	87, 959.18 (403.16)	0.33	87, 0.35 (0.08)	0.037	87, 50.74 (16.76)	0.296	97, 1660.38 (730.76)	0.163	97, 0.42 (0.09)	0.474	97, 79.33 (26.13)	0.165
	Yes	40, 1074.45 (517.08)		40, 0.38 (0.09)		40, 55.07 (20.34)		58, 1786.28 (669.41)		58, 0.43 (0.1)		58, 83.57 (24.17)	
Regular attendance at daycare	No	129, 993.96 (440.3)	0.915	129, 0.36 (0.08)	0.23	129, 52.13 (17.87)	0.936	157, 1703.51 (708.7)	0.565	157, 0.42 (0.09)	0.425	157, 80.79 (25.57)	0.716
	Yes	1, 873 (NA)		1, 0.27 (NA)		1, 47.54 (NA)		4, 1888 (801.89)		4, 0.46 (0.05)		4, 85.64 (28.72)	
Central air conditioner in residence	No	55, 1073.44 (421.36)	0.018	55, 0.38 (0.07)	0.013	55, 55.16 (17.15)	0.037	64, 1862.59 (736.4)	0.043	64, 0.45 (0.07)	0.016	64, 86.31 (24.42)	0.048
	Yes	75, 934.07 (444.56)		75, 0.34 (0.09)		75, 49.84 (18.06)		97, 1606.15 (674.8)		97, 0.41 (0.09)		97, 77.35 (25.8)	
Clothes dryer in residence	No	16, 1216.81 (363.5)	0.004	16, 0.41 (0.06)	0.001	16, 61.11 (16.19)	0.009	21, 1800.24 (693.61)	0.322	21, 0.44 (0.08)	0.471	21, 83.68 (25.97)	0.429
	Yes	114, 961.62 (440.57)		114, 0.35 (0.08)		114, 50.83 (17.72)		140, 1694.27 (712.61)		140, 0.42 (0.09)		140, 80.5 (25.58)	
Use gas cooking stove	No	44, 971.7 (494.36)	0.392	44, 0.35 (0.1)	0.725	44, 51.17 (19.99)	0.372	51, 1765.04 (668.66)	0.302	51, 0.43 (0.08)	0.326	51, 82.73 (24.8)	0.442
	Yes	86, 1003.94 (410.02)		86, 0.36 (0.07)		86, 52.56 (16.69)		110, 1681.69 (728.3)		110, 0.42 (0.09)		110, 80.07 (25.99)	
Regular use of air filter	No	65, 1036.68 (451.44)	0.197	65, 0.36 (0.08)	0.177	65, 54.34 (18.26)	0.104	79, 1885.67 (700.21)	0.001	79, 0.44 (0.08)	0.001	79, 87.92 (24.9)	<0.001
	Yes	63, 936.32 (415.78)		63, 0.35 (0.08)		63, 49.39 (16.88)		77, 1490.6 (642.02)		77, 0.4 (0.09)		77, 72.3 (23.26)	
Year residence built	=< 1950	50, 985.84 (396.57)	0.236	50, 0.36 (0.08)	0.053	50, 51.48 (15.96)	0.291	65, 1723.35 (757.68)	0.988	65, 0.42 (0.1)	0.799	65, 81.5 (27.56)	0.966
	1950-1979	56, 1009.91 (444.83)		56, 0.36 (0.07)		56, 52.96 (18.31)		67, 1698.67 (698.06)		67, 0.43 (0.08)		67, 80.88 (25.28)	
	1979-2012	20, 883 (445.74)		20, 0.32 (0.1)		20, 47.26 (17.05)		23, 1668.65 (652.59)		23, 0.41 (0.09)		23, 79.4 (23.04)	
**Environment – ISV**													

		Neonates						Infants					
		Richness		Evenness		Diversity		Richness		Evenness		Diversity	
Factor		N, Mean (SD)	P*	N, Mean (SD)	P	N, Mean (SD)	P	N, Mean (SD)	P	N, Mean (SD)	P	N, Mean (SD)	P
Indoor pet(s)	Neither							120, 1760.62 (711.58)	0.349	120, 0.43 (0.09)	0.146	120, 82.66 (25.4)	0.337
	Dog only							25, 1735.92 (788.66)		25, 0.44 (0.08)		25, 82.55 (27.99)	
	Cat only							12, 1432.33 (569.15)		12, 0.4 (0.07)		12, 72.17 (23.26)	
	Both							10, 1454.3 (686.39)		10, 0.39 (0.1)		10, 69.58 (23.03)	
ETS	No							139, 1708.65 (728.18)	0.729	139, 0.42 (0.09)	0.537	139, 80.94 (26.21)	0.718
	Yes							29, 1726.83 (654.46)		29, 0.43 (0.09)		29, 81.45 (22.64)	
Endotoxin - Mother's mattress ł	---							159, 0.1	0.195	159, 0.1	0.203	159, 0.09	0.257
Endotoxin - Mother's bedroom floor ł	---							158, 0.18	0.021	158, 0.25	0.002	158, 0.19	0.018
Endotoxin - Baby's mattress ł	---							164, 0.09	0.251	164, 0.04	0.573	164, 0.1	0.21
Endotoxin - Baby's bedroom floor ł	---							161, 0.22	0.005	161, 0.26	0.001	161, 0.24	0.002
Endotoxin - living room floor ł	---							162, 0.17	0.032	162, 0.2	0.011	162, 0.16	0.039
Mom smoked	No							153, 1711.29 (735.63)	0.718	153, 0.42 (0.09)	0.664	153, 80.91 (26.31)	0.621
	Yes							15, 1716.8 (456.72)		15, 0.44 (0.06)		15, 82.16 (16.68)	
Detectable Can f 1	No							64, 1663.94 (645)	0.743	64, 0.41 (0.09)	0.219	64, 80.01 (24.46)	0.904
	Yes							102, 1757.36 (753.53)		102, 0.43 (0.08)		102, 82.17 (26.31)	
Detectable Fel d 1	No							40, 1816.6 (818.15)	0.392	40, 0.44 (0.1)	0.298	40, 84.79 (30.65)	0.445
	Yes							127, 1684.91 (678.07)		127, 0.42 (0.08)		127, 80.02 (23.78)	
Detectable Der f 1	No							90, 1721.59 (730.65)	0.954	90, 0.43 (0.08)	0.854	90, 81.44 (25.91)	0.958
	Yes							77, 1710.44 (698.18)		77, 0.42 (0.09)		77, 80.85 (25.35)	

Factor		Neonates Richness N, Mean (SD)	P*	Neonates Evenness N, Mean (SD)	P	Neonates Diversity N, Mean (SD)	P	Infants Richness N, Mean (SD)	P	Infants Evenness N, Mean (SD)	P	Infants Diversity N, Mean (SD)	P
Regular attendance at daycare	No							97, 1629.4 (742.59)	0.031	97, 0.41 (0.09)	0.032	97, 77.38 (26.19)	0.019
	Yes							71, 1824.34 (662.02)		71, 0.44 (0.08)		71, 86 (23.97)	
Central air conditioner in residence	No							69, 1842.14 (729.85)	0.056	69, 0.45 (0.07)	0.007	69, 86.43 (25.04)	0.029
	Yes							99, 1620.93 (692.25)		99, 0.41 (0.09)		99, 77.26 (25.37)	
Clothes dryer in residence	No							23, 2010.61 (885.15)	0.09	23, 0.47 (0.08)	0.005	23, 90.14 (30.42)	0.139
	Yes							144, 1663.01 (676.88)		144, 0.42 (0.09)		144, 79.5 (24.58)	
Use gas cooking stove	No							49, 1767.69 (721.49)	0.444	49, 0.43 (0.09)	0.772	49, 82.88 (26.08)	0.521
	Yes							119, 1688.76 (712.91)		119, 0.42 (0.09)		119, 80.26 (25.42)	
Regular use of air filter	No							82, 1838.91 (731.04)	0.018	82, 0.44 (0.08)	0.037	82, 85.96 (25.75)	0.012
	Yes							79, 1555.73 (687.26)		79, 0.41 (0.09)		79, 74.96 (24.73)	
Year residence built	=< 1950							44, 1724.8 (801.4)	0.573	44, 0.43 (0.09)	0.281	44, 81.01 (27.91)	0.507
	1950-1979							98, 1743.09 (711.84)		98, 0.43 (0.09)		98, 82.32 (25.95)	
	1979-2012							17, 1536.88 (562.91)		17, 0.39 (0.09)		17, 75.02 (19.77)	
**Specimen Characteristics**													
Age at stool collection (days) ł	---	130, 0.27	0.002	130, 0.21	0.019	130, 0.25	0.004	168, 0.25	0.001	168, 0.27	<0.001	168, 0.27	<0.001
Season stool collected	Winter	38, 1001.97 (469.08)	0.04	38, 0.35 (0.09)	0.182	38, 52.79 (19.49)	0.08	47, 1733.96 (680.15)	0.869	47, 0.43 (0.09)	0.874	47, 82.21 (24.54)	0.788
	Spring	27, 876.56 (324.27)		27, 0.34 (0.08)		27, 47.65 (13.72)		59, 1684.41 (772.57)		59, 0.42 (0.1)		59, 79.78 (26.41)	
	Summer	33, 914.85 (396.51)		33, 0.35 (0.08)		33, 48.81 (15.51)		32, 1754.5 (760.68)		32, 0.44 (0.08)		32, 82.54 (27.91)	
	Fall	32, 1161.31 (490.08)		32, 0.39 (0.07)		32, 58.4 (19.75)		30, 1685.33 (621.23)		30, 0.42 (0.07)		30, 80.02 (23.91)	

Abbreviations: BMI, body mass index; ETS, environmental tobacco smoke; NSV, neonatal study visit at 1-month of age; ISV, infant study visit at 6-months of age.
*Wilcoxon Rank Sum or Kruskal-Wallis
ł Spearman’s correlation and corresponding p-value reported for continuous variables

See Excel spreadsheet for Supplementary Table S5
